# A constructive approach for discovering new drug leads: Using a kernel methodology for the inverse-QSAR problem

**DOI:** 10.1186/1758-2946-1-4

**Published:** 2009-04-28

**Authors:** William WL Wong, Forbes J Burkowski

**Affiliations:** The David R. Cheriton School of Computer Science, University of Waterloo, Waterloo, Ontario N2L 3G1 Canada

**Keywords:** Feature Space, Input Space, Molecular Descriptor, Reproduce Kernel Hilbert Space, Outgoing Edge

## Abstract

**Background:**

The inverse-QSAR problem seeks to find a new molecular descriptor from which one can recover the structure of a molecule that possess a desired activity or property. Surprisingly, there are very few papers providing solutions to this problem. It is a difficult problem because the molecular descriptors involved with the inverse-QSAR algorithm must adequately address the forward QSAR problem for a given biological activity if the subsequent recovery phase is to be meaningful. In addition, one should be able to construct a feasible molecule from such a descriptor. The difficulty of recovering the molecule from its descriptor is the major limitation of most inverse-QSAR methods.

**Results:**

In this paper, we describe the reversibility of our previously reported descriptor, the vector space model molecular descriptor (VSMMD) based on a vector space model that is suitable for kernel studies in QSAR modeling. Our inverse-QSAR approach can be described using five steps: (1) generate the VSMMD for the compounds in the training set; (2) map the VSMMD in the input space to the kernel feature space using an appropriate kernel function; (3) design or generate a new point in the kernel feature space using a kernel feature space algorithm; (4) map the feature space point back to the input space of descriptors using a pre-image approximation algorithm; (5) build the molecular structure template using our VSMMD molecule recovery algorithm.

**Conclusion:**

The empirical results reported in this paper show that our strategy of using kernel methodology for an inverse-Quantitative Structure-Activity Relationship is sufficiently powerful to find a meaningful solution for practical problems.

**Electronic supplementary material:**

The online version of this article (doi:10.1186/1758-2946-1-4) contains supplementary material, which is available to authorized users.

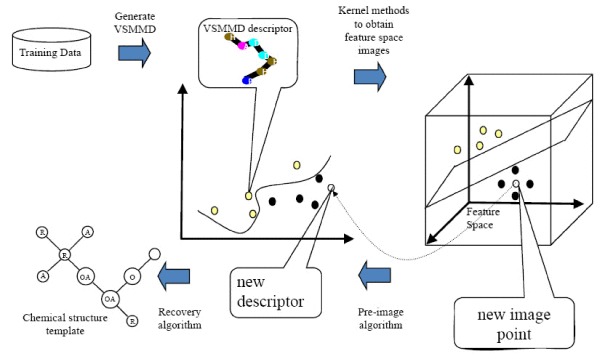

## Background

The structural conformation and physicochemical properties of both the ligand and its receptor site determine the level of binding affinity that is observed in such an interaction. If the structural properties of the receptor site are known (for example, there is crystallographic data) then techniques involving approximations of potential functions can be applied to estimate or at least compare binding affinities of various ligands [1]. When this information is sparse or not available, as is the case for many membrane proteins, it becomes necessary to estimate affinities using only the properties of the ligand. This ligand-based prediction strategy is often used in applications such as virtual screening of molecular databases in a drug discovery procedure.

In a more general setting we strive to establish the quantitative dependency between the molecular properties of a ligand and its binding affinity. To restate this goal using current terminology: we want to analyze the Quantitative Structure-Activity Relationship (QSAR) of the ligand with respect to this type of receptor. A common approach for a QSAR analysis is the use of a machine learning strategy that processes sample data to learn a function that will predict binding affinities. The input of such a function is a descriptor: a vector of molecular properties [2] that characterize the ligand. These vector entries may be physicochemical properties, for example, molecular weight, surface area, orbital energies, or they may describe topological indices that encode features such as the properties of individual atoms and bonds. Topological indices can be rapidly computed and have been validated by a variety of experiments investigating the correlation of structure and biological activities.

The inverse-QSAR problem seeks to find a new molecular descriptor from which one can recover the structure of a molecule that possess a desired activity or property. Surprisingly, there are relatively few papers providing solutions to this problem [3]. Lewis [4] has investigated automated strategies for working with fragment based QSAR-driven transforms that are applied to known molecules with the objective of providing new and promising drug leads. Brown *et al*. [5] have studied the inverse QSPR problem using multi-objective optimization. They used an interesting partial least squares (PLS) related algorithm in the design of novel chemical entities (NCEs) that satisfy a given property range or objective. The physical properties considered in their paper were mean molecular polarizability and aqueous solubility. A problem with the opposite emphasis has also been investigated: Masek *et al*. [6] considered sharing chemical information that is encoded in such a way as to *prevent* recovery of the original molecule (a strategy used in the facilitation of prediction software while protecting intellectual property rights).

In general, the inverse-QSAR problem is difficult because the molecular descriptors involved with the inverse-QSAR algorithm must adequately address the forward QSAR problem for a given biological activity if the subsequent recovery phase is to be meaningful. In addition, one should be able to construct a feasible molecule from such a descriptor. The difficulty of recovering the molecule from its descriptor is the major limitation of most inverse-QSAR methods.

Most of the proposed techniques are stochastic in nature [7–9], however, a limited number of deterministic approaches have been developed including the approach of Kier and Hall [10–13] based on a count of paths, and an approach based on signature descriptors (see Faulon *et al*. [14–17]).

The key to an effective method lies in the use of a descriptor that facilitates the reconstruction of the corresponding molecular structure. Ideally, such a descriptor should be informative, have good correlative abilities in QSAR applications, and most importantly, be computationally efficient. A computationally efficient descriptor should have a low degeneracy, that is, it should lead to a limited number of solutions when a molecular recovery algorithm is applied.

Currently, kernel methods are popular tools in QSAR modeling and are used to predict attributes such as activity towards a therapeutic target, ADMET properties (absorption, distribution, metabolism, excretion, and toxic effects), and adverse drug reactions. Various kernel methods based on different molecular representations have been proposed for QSAR modelling [18]. They include the SMILES string kernel [19], graph kernels [20–22] and a pharmacophore kernel [23]. However, none of these kernel methods have been used for the inverse-QSAR problem.

In this paper, we investigate the reversibility of our previously reported descriptor, the vector space model molecular descriptor (VSMMD) [24]. VSMMD is based on a vector space model that is suitable for kernel studies in QSAR modeling. Our approach to the inverse-QSAR problem consists of first deriving a new image point in the kernel feature space and then finding the corresponding pre-image descriptor in the input space. Then, we use a recovery algorithm to generate a chemical structure *template* to be used in the specification of new drug candidates. Template formats will vary with respect to their specificity. Depending on the nature of the recovery process, a template may specify a unique molecule or a family of molecules. In the latter case, molecules meeting the specification could be obtained by means of high throughput screening. In the "Methods" section, we provide a detailed description of our inverse-QSAR approach using our VSMMD approach. In the "Results" section, we present the experimental results of our descriptors in the vector space setting.

## Methods

**Figure 1:**
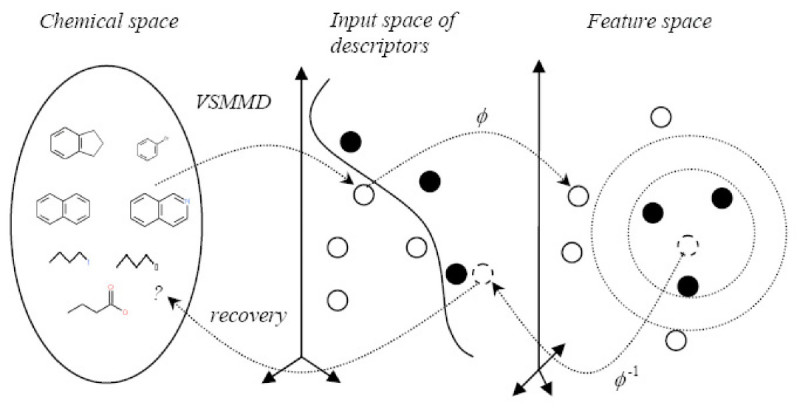
**Overall concept for the VSMMD inverse-QSAR approach**.

Our inverse-QSAR approach can be described in five steps. The first two steps perform a QSAR analysis. In the first step, we generate a VSMMD for each compound in the training set. Then, in the second step, we use a kernel function to map each VSMMD to a feature space typically used for classification. The third step is to design or to generate a new point in the kernel feature space using a kernel feature space algorithm (e.g. the center of highly active compounds). In the fourth step, we map this point from the feature space back to the input space using a pre-image approximation algorithm. In the last step, the molecular structure template will be built by our VSMMD molecule recovery algorithm. Figure 1 illustrates the overall processing.

### Vector Space Model Molecular Descriptor (VSMMD)

The vector space model molecular descriptor (VSMMD) [24] can be categorized as belonging to the constitutional descriptors that provide feature counts related to the two dimensional structure of a molecule. Functionally, our descriptor bears a resemblance to various other descriptors such as reduced graphs (see Gillet [25]) and circular fragments (Glenn *et al*. [26]). One may also see our fragment oriented approach as somewhat reminiscent of the path count strategy of Kier and Hall [10–13]. In the setting of inverse-QSAR, we used VSMMD because we were familiar with its capabilities and we had software applications to do the generation of the descriptors. The primary significance of the current study is not the formulation of a new and competitive descriptor but rather the development of strategies to facilitate the reverse engineering workflow that one needs to go from feature space point back to the specification of a new ligand.

**Figure 2:**
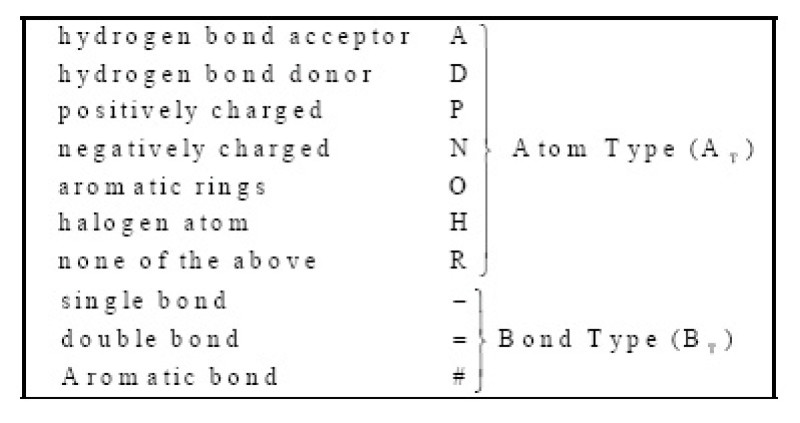
**Labels for atoms and bonds in a molecule**.

The first step in constructing the VSMMD is to identify the physicochemical properties of each atom in a molecule. Specifically, we affix labels to atoms and bonds as specified in Figure 2. It should be noted that triple bonds would also be labelled as "=". This helps to reduce the combinatorial explosion of components in the descriptor at the expense of introducing some degeneracy. Since the occurrence of triple bonds is rather infrequent, we contend that this low level of degeneracy is much more acceptable than the drastic and unwanted increase in component count.

The VSMMD strategy is based on the extraction of molecular fragments that are comprised of small sets of bonded atoms. The atom count for a fragment is at least two and at most *c*, where *c* is some pre-specified value such as 2, 3, or 4.

**Figure 3:**
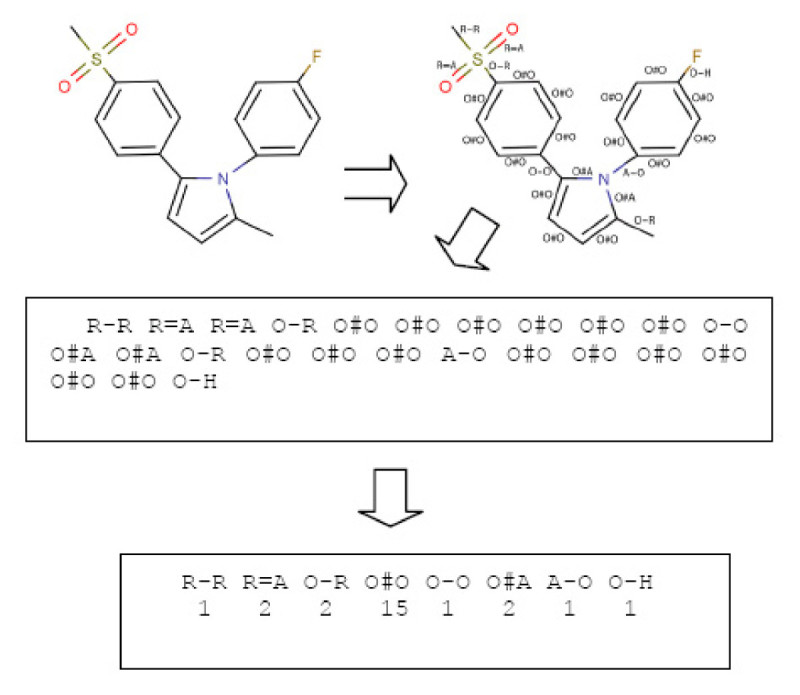
**Processing steps for the VSMMD**.

To illustrate the processing of a molecule we describe the steps that are taken in the processing of a molecule (atom count for a fragment limited to 2). Figure 3 shows one of the pyrrole compounds that is a member of the data set used in reference [27].

The algorithm goes through the following steps:

1. The atoms and bonds are labelled as prescribed by Figure 2.
2. The molecular descriptor is created by extracting from the molecule a complete set of small fragments.
3. Frequency counts are evaluated for all the fragments so that a multi-set or bag of fragments can be generated

When these steps are completed, the multi-set counts are placed into a vector that has a position for each of the different possible fragments recorded in the dictionary. Note that all molecules end up with descriptors of the same length. If fragment size is limited to 2 atoms this vector would have a dimensionality of 7*7*3 = 147.

### VSM and VSMMD compared

The motivation for the VSMMD descriptor comes from the "bag-of-words" approach [28] that is based on the vector space model (VSM) used in information retrieval. Roughly speaking, the atom fragments extracted in the VSMMD process corresponds to the document words and phrases extracted in the VSM. The molecule is then analogous to a text document and much of the analysis used in the bag-of-words strategy can be brought over to the VSMMD setting.

In practice, we have found that the success of VSMMD is greatly enhanced by utilizing fragments that contain more than two atoms, for example, *c* = 3 or *c* = 4. This adoption of a higher level of structure corresponds to the incorporation of phrase structures in the VSM. From a molecular perspective, the larger fragment will incorporate information related to rotation around a single bond. As the value of *c* increases there is a point of diminished returns due to the combinatorial explosion of fragment possibilities. To help reduce this "curse of dimensionality", we can remove the vector entries that have been observed to have frequency counts equal to zero across all molecules under consideration.

In the VSM, a language dictionary can be defined using some permanent predefined set of words. In our model, according to the encoding scheme, the dictionary will be the collection of all possible atom type (A_T_) and bond type (B_T_) labelled graphs that arise from molecular fragments that are restricted to having atom counts of 2, 3, or 4. Table 1 shows the general format of our dictionary with *c* limited to 4. The last entry of this table represents a four atom fragment in which a central atom is bonded to three other atoms.

**Table 1:** General format of the fragment dictionary (A_T_ = Atom Type, B_T_ = Bond Type).

Type ID	General Fragment Type	Atom Count
1)	A_T_ B_T_ A_T_	2
2)	A_T_ B_T_ A_T_ B_T_ A_T_	3
3)	A_T_ B_T_ A_T_ B_T_ A_T_ B_T_ A_T_	4
4)	A_T_ B_T_ A_T_ B_T_ A_T_	4
	B_T_	
	A_T_	

In the most general case, a molecular descriptor is represented by a bag of fragments, each fragment corresponding to an entry in the dictionary.

### Analysis based on VSMMD

We now describe the notation and mathematical setting used in VSMMD. For each molecule *i*, a molecular descriptor *d*_*i*_can be generated. We represent the descriptor as a column vector in an *m* dimensional space using the mapping:
1

where *q*(*f*_l_, *d*_*i*_) is the frequency of the fragment *f*_l_ in the descriptor *d*_*i*_.

The use of the linear kernel *ɸ*_*L*_(·) in this last equation is deliberate since we want to view this mapping as the type of kernel function that is used in the bag-of-words strategy described by Shawe-Taylor and Cristianini [28]. Via this mapping, each molecular descriptor is taken over to an *m*-dimensional vector, where *m* is the size of the dictionary. Although *m* could be very large, the typical vector generated in this way is usually quite sparse (just as vectors in the VSM are sparse).

Working with *n* molecules, we can apply the mapping repeatedly to generate a succession of column vectors: *ɸ*_*L*_(*d*_1_),*ɸ*_*L*_(*d*_2_),⋯,*ɸ*_*L*_(*d*_*n*_). Computation of the vector space kernel is done by calculating the fragment-descriptor matrix *F* with rows indexed by the fragments and columns indexed by the descriptors:
2

The entry at position (*i, j*) gives the frequency of fragment *f*_*i*_in molecule *j*. Subsequently, we can create the kernel matrix as:
3

corresponding to the vector space inner product
4

In our forward-QSAR experiments [24], the nonlinear Gaussian kernel [28]
5

gave the best results. It provides a rich hypothesis space and is often used in machine learning studies. One parameter, *σ*, had to be evaluated through cross-validation.

With the Gaussian vector space kernel, we can apply a kernel-based method to generate a predictor of any one of several biological activities, for example: ADMET properties or affinity of ligands used as therapeutic agents. In our previous studies [24], we gave empirical evidence to demonstrate that VSMMD can capture important chemical information allowing it to perform very well in both forward-QSAR studies and the determination of binding mode information.

### The Reproducing Kernel Hilbert Space (RKHS)

Kernel-based learning algorithms work by embedding the data into a Hilbert space, often called the feature space followed by a search for linear relations within this Hilbert space. Kernels are functions that can be used to formulate similarity comparisons. They provide a general framework to represent data, subject to certain mathematical conditions. Data are not represented individually by kernels. Instead, data are represented through a set of pair-wise comparisons.

**Figure 4:**
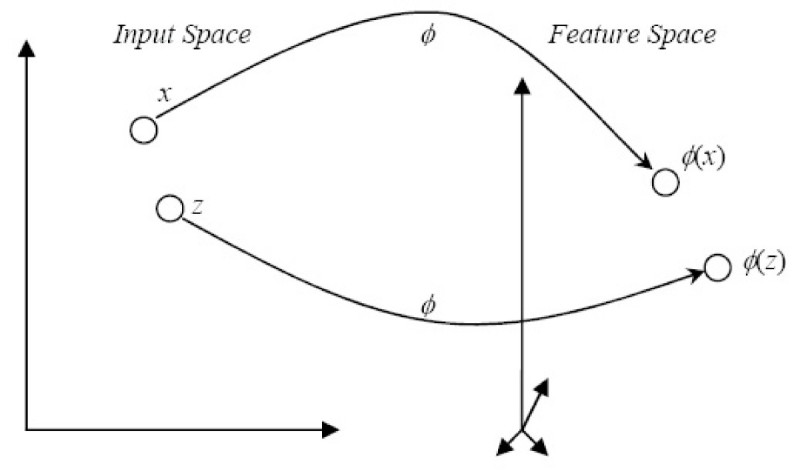
**The implicit function** ***ɸ*** **maps points in the input space over to the feature space**.

More formally, suppose we have *n* training data pairs  where *x*_*i*_∈ *X*. The vector space *X* is referred to as the input space and each *y*_*i*_is considered to be a component in *Y*, the vector of the output values. In the process of machine learning, we want to be able to generalize to previously unseen data points. In the case of binary classification, given some new input *x* ∈ *X*, we want to predict the corresponding *y* ∈ {+ 1, -1}. In other words, we want to choose *y* such that (*x, y*) is in some sense similar to the training examples. In order to achieve this, we require a similarity measures in *X* and in *Y*. Since *y* ∈ {+1, -1}, to find the similarity measure in *Y* is relatively easy. On the other hand, we require a function to measure the similarity in *X*:
6

satisfying, for all *x*, *x*_*i*_∈ *X*: *i* = 1 .. *n*,
7

where *ɸ* maps descriptors into an inner product feature space *FS*. The similarity function *k* is called a kernel, and *ɸ* is its feature map. The feature space *FS* is usually called the reproducing kernel Hilbert space (RKHS) associated with *k*. Figure 4 illustrates the overall mapping concept.

By using a kernel function, the embedding in the Hilbert space is actually performed implicitly, that is by specifying the inner product between each pair of points rather than by giving their coordinates explicitly. This approach has several advantages, the most important being the fact that often the inner product in the embedding space can be computed much more easily using a kernel rather than using the coordinates of the points themselves.

In machine learning, using kernels is a strategy for converting a linear classifier algorithm into a non-linear one by using a non-linear function to map the original observations into a higher-dimensional feature space; this makes a linear classification in the new feature space equivalent to a non-linear classification in the original input space.

For more details about the RKHS, readers can refer to [28].

### Designing Molecules Using a Feature Space Algorithm

Suppose we have a set of *n* molecular descriptors *S*, designated as *S* ={*d*_1_, *d*_2_,⋯,*d*_*n*_} where each *d*_*i*_is in the input space *X*. Let us assume we are using a Gaussian vector space kernel as defined in equation (5). Under this kernel, any point *d*_*i*_∈ *X*, is implicitly mapped to an image *ɸ*(*d*_*i*_) in the feature space *FS*. With this kernel mapping, we can define the set *ɸ*(*S*) = {*ɸ*(*d*_1_), *ɸ*(*d*_2_),⋯,*ɸ*(*d*_*n*_)} ⊂ *FS*.

In this sub-section, we will evaluate various properties of the data set *ɸ*(*S*). We provide a set of elementary algorithms to do various calculations such as distance between two descriptor image points in the feature space.

#### The feature space centroid derived from highly active compounds

The norm of *ɸ*(*d*) is given by:
8

A special case of the norm is the length of the line joining two images *ɸ*(*d*_1_) and *ɸ*(*d*_2_), which can be computed using:
9

The norm described by (9) represents the distance between two descriptor image points in the feature space. We define the centroid *ɸ*_*s*_of the molecule data set *S* in the feature space as:
10

The norm of the centroid can be calculated using only the evaluations of the kernel on the inputs:
11

Note that, the result is the average of the entries in the kernel matrix. The inner product between a descriptor image point *ɸ*(*d*) and the centroid *ɸ*_*s*_is given by:
12

Using equation (9), we can calculate the distance between *ɸ*(*d*) and the centroid *ɸ*_*s*_in the feature space by:
13

**Figure 5:**
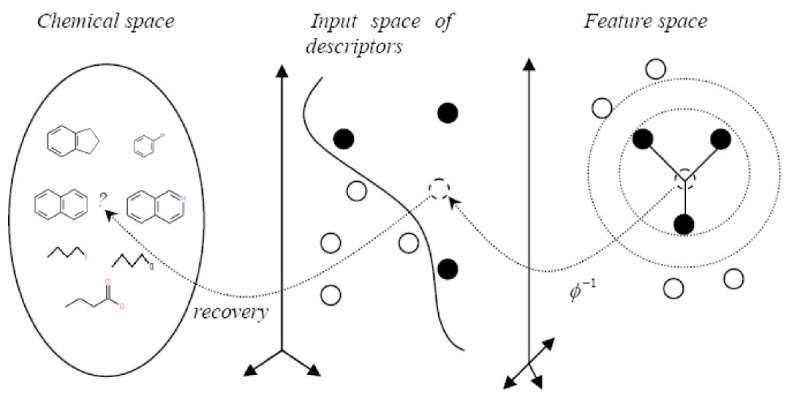
**Deriving a new image in kernel feature space**.

Recall that the kernel-based learning algorithms work by embedding the data into the feature space, and searching for a linear relationship within this feature space. With this linear relationship, it makes sense to derive a new descriptor image using the centroid point of the highly active compound's image points which will share the general properties of all highly active compounds. If the centroid point can be mapped from the feature space back to the input space, we can obtain the descriptor of a new candidate molecule. Figure 5 illustrates this idea. It should be stressed that when a nonlinear kernel (such as the Gaussian kernel) is used, then a point in the feature space bears a nonlinear relationship to the point in the input space. The centroid in the feature space would rarely, if ever, map back to the centroid in the input space. We have chosen to use the centroid in the feature space because there is more assurance of generating a new point in the feature space that in some sense represents a point of reasonable interpolation in this high dimensional space. Picking an arbitrary point in the feature space runs a higher risk of extrapolation which may be difficult to avoid especially when a small training set is spread over the higher dimensional feature space in some rarefied manner.

The inverse in the input space is called the pre-image. We will discuss pertinent details in the pre-image problem subsection to follow.

There are several studies that use a feature space centroid to generate new data. Kwok and his colleagues used a feature space centroid to generate a new data point for hand-written digit recognition [29] and speech processing [30]. In both applications, the pre-image has been shown to be robust and meaningful. In the next subsection we describe another strategy for the derivation of a new feature space point

#### Minimum enclosing and maximum excluding hyperspheres

In the last subsection, we derived a new descriptor image point using the centroid of feature space images derived from the highly active compounds. In this subsection, we use the highly active compounds to derive two hyperspheres with the same center. The center of the hyperspheres is then mapped back from the feature space to the input space to generate the descriptor of a new candidate molecule.

Suppose we can identify a subset *G* ⊆ *S* where *G* contains the descriptors of molecules in the chemical space with the highest activity. We let |*G*| represent the number of descriptors in *G*. In an ideal situation, the feature space images of *G* will be spherically separable from all the other descriptor images mapped over from *S*. With this assumption, we can derive two hyperspheres, sharing the same center *a*, such that the images of all descriptors derived from highly active molecules are enclosed by the inner hypersphere *H*_1_ and all the remaining images are excluded by the outer hypersphere *H*_2_. Let *r*_1_ be the radius of the inner hypersphere and let *r*_2_ be the radius of the outer hypersphere. Consequently, we have:
14

**Figure 6:**
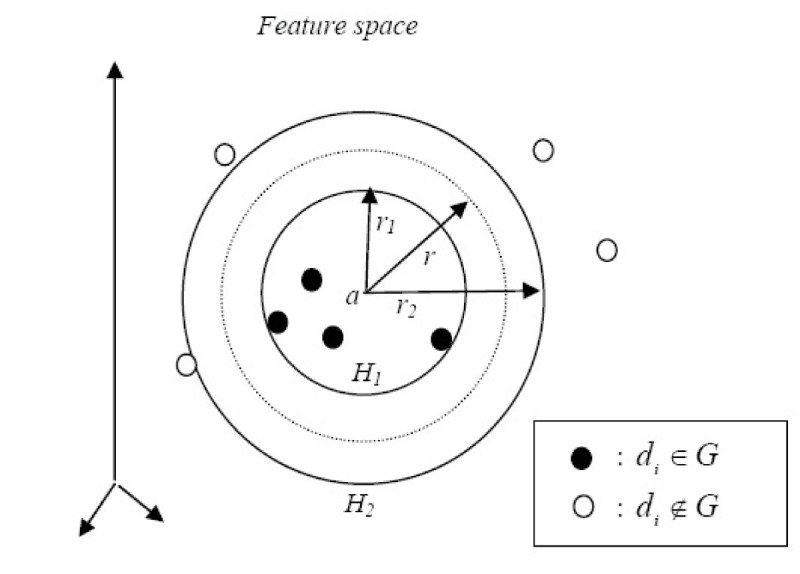
**Minimum enclosing and maximum excluding hyperspheres in the feature space**.

Figure 6 illustrates this idea.

Following the development of Liu and Zheng's minimum enclosing and maximum excluding machine (MEMEM) [31], we want the inner hypersphere *H*_1_ as small as possible for a good description of the highly active class. In the meantime, we want the outer hypersphere *H*_2_ as large as possible. In other words, we try to maximize the area between two hyperspheres *H*_2_ and *H*_1_. Note that the area between two hyperspheres *H*_2_ and *H*_1_ is proportional to the quantity . Let  and , we can formulate the objective function to have  as small as possible and Δ*r*^2^ as large as possible by minimizing the quantity , or equivalently
15

Replacing the constant  by *η*, which controls the trade off between the importance of the inner hypersphere and the outer hypersphere, the objective function will become:
16

Our analysis will require Lagrange multipliers *α*_*i*_and labels *y*_*i*_such that
17

The dual problem of equation (16) can be obtained [31] as:
18

In practice, the data may not be separable in this fashion. By introducing slack variables *ζ* ≥ 0, equation (16) becomes:
19

By using equation (19), we allow some negative samples inside the inner hypersphere and some positive samples outside the outer hypersphere. The corresponding dual problem of equation (19) obtained by [31] is:
20

where *β* is the Lagrange multiplier associated with the constraint Δ*r*^2^ ≥ 0, and *C* is some constant to be determined with a validation data set. The center *a* of the hyperspheres is then mapped back from the feature space to the input space to generate the descriptor of a new candidate molecule.

### The Pre-image Problem

**Figure 7:**
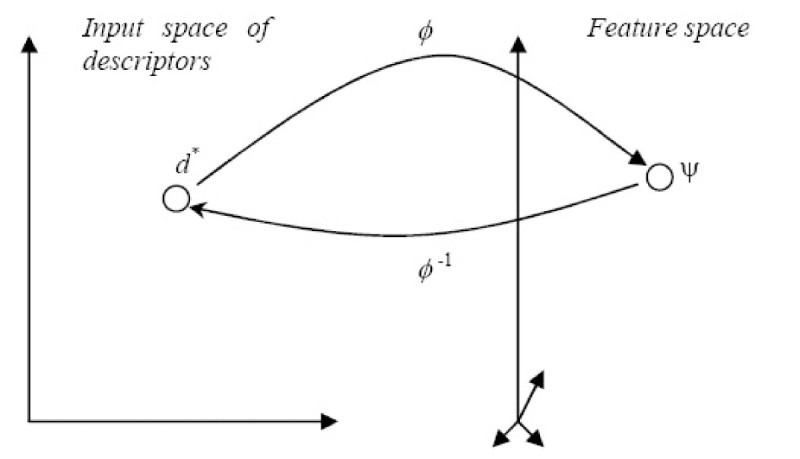
**The pre-image problem**.

In RKHS subsection, we illustrated how a point in the input space is mapped to the feature space via the implicit function *ɸ*. In this subsection, we are interested in finding how a point in the feature space can be mapped back to the input space. Formally, this is called the pre-image problem of reconstructing patterns from their representation in feature space (see Figure 7).

Let Ψ be a point in the reproducing kernel Hilbert space (RKHS) *FS*. The pre-image of Ψ ∈ *F* is a point d* ∈ *X* (the original input space). Formally,
21

The problem of finding the pre-image d* is equivalent to the problem of finding the inverse of *ɸ* defined in equation (21):
22

However, the problem of finding *ɸ*^-1^(·) is typically an ill-posed problem. A problem is said to be ill-posed if the solution is not unique, does not exist, or is not a continuous function of the data [32].

One possible way to overcome this problem is to look for  an approximation of the pre-image such that *ɸ*() is as close as possible to Ψ. Formally, we search for  ∈ *X*, such that
23

Typically, we will have Ψ defined as some linear combination of implicit mappings from the input space. Consequently, expanding equation (23), we get:
24

which can be rewritten as:
25

Using equation (25), an inversion problem turns out to be an optimization problem. There are several algorithms that attempt to solve this optimization problem. Schölkopf *et al*. [33] proposed an iterative fixed point algorithm strategy. Kwok and Tsang [28] proposed another method that exploits the correspondence between distances in the input space and the feature space. Alternatively, a standard gradient optimization method can be used to find an approximation of the pre-image [33]. Note that all these methods are only guaranteed to find a local optimum.

**Figure 8:**
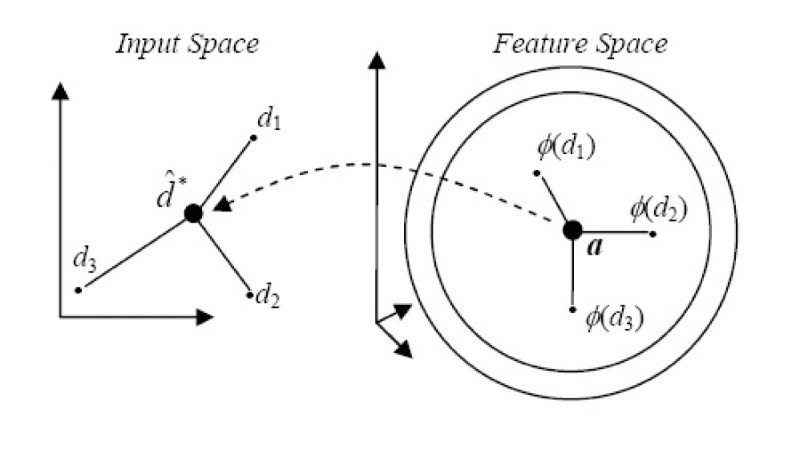
**Kwok and Tsang pre-image strategy**.


In this paper, we are going to follow Kwok and Tsang [28] and use their approach to approximate the pre-image. Their algorithm is based on the notion of distance constraints. They assume that there exists a simple relationship between distances in the input space and distances in feature space. Figure 8 illustrates these relationships. Suppose we have derived the center *a* of the hyperspheres using equation (19). It was shown that the center *a* of the hyperspheres is a linear combination of the training samples [31] i.e.: . Recall that *η* is defined in equation (16). The norm of the center can be calculated using only the evaluations of the kernel on the training sample inputs:

26


For each training sample *d*_*i*_we can derive  representing the square of the distance between the training sample image *d*_*i*_and the center *a* of the hyperspheres:
27

Using the Gaussian kernel as specified in equation (5) and an observation made by Kwok and Tsang [29], the corresponding input space distance *dist*_*a*, *i*_, between the center and training sample *d*_*i*_can be found using:
28

To speed up the algorithm (as observed by Kwok and Tsang [29]), only the *p* closest training sample images of *a* will be considered. From (27), we can identify the *p* closest neighbours of *a* in the feature space. Using (28), we can convert these *p* closest neighbour distances in the feature space to their corresponding input space distances. Let **b** be the vector representing these input space distances with
29

Their corresponding descriptors in the input space are *d*_1_, *d*_2_,⋯,*d*_*p*_∈ ℝ^*m*^, and the centroid is defined as . Let *D* = [*d*_1_, *d*_2_,⋯,*d*_*p*_] be an *m* × *p* matrix. To establish the centroid at the origin, we let
30

where *I* is a *p* × *p* identity matrix, and **1** is a *p* dimensional vector with each component equal to 1.

Assuming matrix *D* is of rank *q*, we obtain the singular value decomposition (SVD) of *A*^*T*^as:
31

where *U* = [*u*_1_, *u*_2_,⋯,*u*_*q*_] is a *m* × *q* matrix with orthonormal columns composed of the *u*_*i*_'s, and *Z* = [*z*_1_, *z*_2_,⋯,*z*_*p*_] is a *q* × *p* matrix with the *i*-th column *z*_*i*_being the projection of *d*_*i*_onto the *u*_*j*_vectors such that the squared distance of *d*_*i*_to the origin is equal to ||*z*_*i*_||^2^. Letting **b**_0_ = [||*z*_1_||^2^, ||*z*_2_||^2^,⋯,||*z*_*p*_||^2^]^*T*^the pre-image  of the center *a* can be obtained by:
32

### The Need for a Nonlinear Kernel

The nonlinear implicit mapping provided by the kernel operation allows us to generate an inner product in the feature space by computing a kernel function that has arguments taken from the input space. More significantly, when a nonlinear kernel is used, linear operations in the feature space correspond to nonlinear operations in the input space. This is important because the nonlinear mapping will involve various cross products of components within a vector of the input space. As a consequence, linear structures within the feature space correspond to nonlinear or "warped" structures in the input space.

To illustrate this, we ran a small experiment with the COX2 training set (described below in the Results section): As described earlier, the new feature space point *a*, generated by extracting the center of the enclosing hypersphere, was mapped back to the input space to get its pre-image . We then formed the set of points *S*_*fe*_in the input space (taken from the training set) that produced the ten closest neighbours of *a* under the kernel mapping. This set *S*_*fe*_was compared with the set *S*_*in*_containing the 10 closest neighbours of  in the input space (these neighbours derived using the Euclidean metric). Because of the warping effect, pre-images of close neighbours in the feature space are not necessarily the closest neighbours of the pre-image  in the input space, in fact, the intersection of *S*_*in*_and *S*_*fe*_is only 3 descriptors. More significant: the average affinity of molecules in *S*_*in*_is 8.03 while the average affinity of molecules in *S*_*fe*_is 8.73. This provides empirical evidence that the nonlinear mapping provided by the kernel function helps us to select input space descriptors that are more significant when considering their corresponding affinities.

### Recovering the Molecule

In order to solve the recovery problem for chemical structures, we have to investigate a way to derive a graph representing the 2D structure of a molecule that has  as its descriptor. There are several related studies that attempt to find such a graph, see for example Bakir *et al*. [34], who worked with a stochastic search algorithm. However, this recovery problem is not well studied from a computational viewpoint. Tatsuya and Fukagawa [35, 36] proposed a dynamic programming algorithm for inferring a chemical structure from a descriptor. However, the algorithms are not practical for a large data set. Previous studies focused on creating a real chemical structure, which defined too many constraints on the problem due to the complexity of chemical structure. In our study, we do not attempt to recover a real chemical structure; instead, we generate a chemical structure template with physiochemical properties only. This simplifies the problem and makes it practical for real data sets.

### Reversible VSMMD

**Figure 9:**
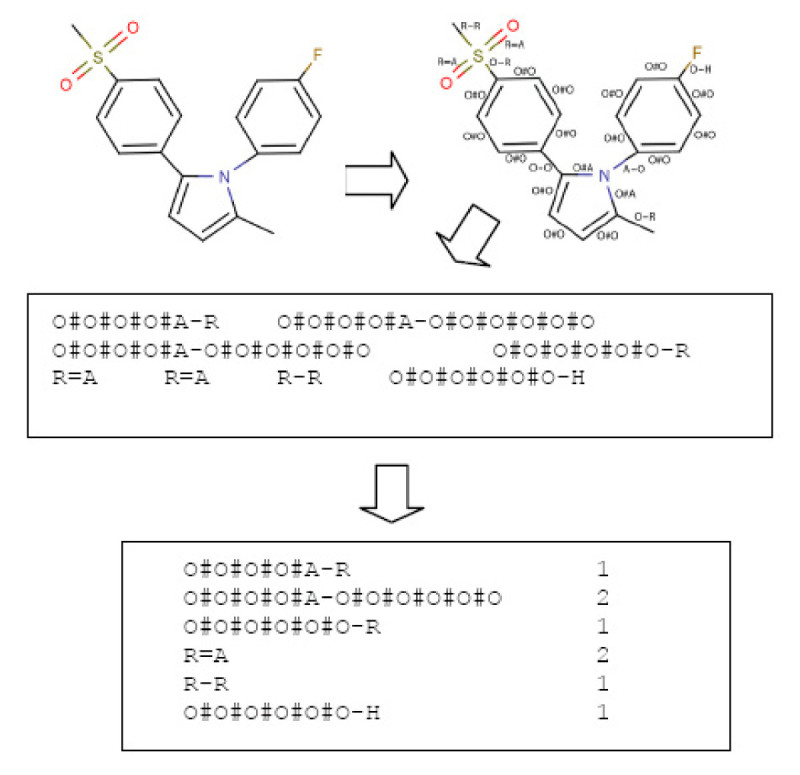
**Simplified VSMMD with an aromatic ring treated as a super atom**.

For illustration and without loss of generality, we will assume that the atom count associated with the VSMMD is two, and we further assume all the aromatic rings are replaced by "super atoms" containing all the rings' physicochemical properties. Figure 9 illustrates a simplified VSMMD using the same example as in Figure 3.

**Figure 10:**
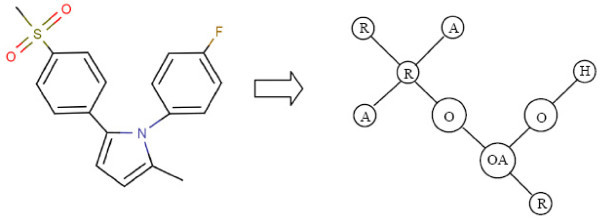
**An example of a chemical structure template**. Note: We use "O" to denote O#O#O#O#O#O and 'OA' to denote O#O#O#O#A.

From Figure 9, we observe that the VSMMD model contains only the physiochemical properties of the chemical structure. As a result, for the recovery problem, we do not attempt to recover the entire chemical structure. Instead, we attempt to generate a chemical structure template with physiochemical properties only. A chemical structure template converted from the molecule shown in Figure 9 is illustrated in Figure 10.

In the next subsection, we define the notion of a structure template and show how it can be derived.

#### Forming the De Bruijn graph

If we consider a molecule to be comprised of molecular fragments then it is clear that there is a hierarchical organization of these fragments. A linear fragment with an atom count of three can be seen as containing two smaller fragments each with an atom count of two and of course the two fragments overlap in the central atom. If we restrict a fragment to have an atom count of two, then it will contain two elementary fragments, namely two atoms, each labelled with their atom types.

Informally: The purpose of a De Bruijn graph is to provide a data structure that shows how small fragments combine to build larger fragments. Since we wish to handle ring structures using the simplification of a "super atom", we will abuse these concepts slightly and consider the fragments under discussion to be fragments within a template as described in the previous subsection.

Suppose we are dealing with fragments that have an atom count designated as *f*_*a*_. The De Bruijn graph *D* is constructed in the following way: We provide a vertex for each fragment that has an atom count equal to *f*_*a*_- 1. In our simplified case, *f*_*a*_= 2 and each vertex will represent an atom labelled with a physicochemical property. We then add a bi-directional edge from vertex *a* to vertex *b* if the fragments associated with these vertices are within a larger fragment with atom count equal to *f*_*a*_. Each edge is weighted with a value representing the number of times that this larger type of fragment occurs in the template.

**Figure 11:**
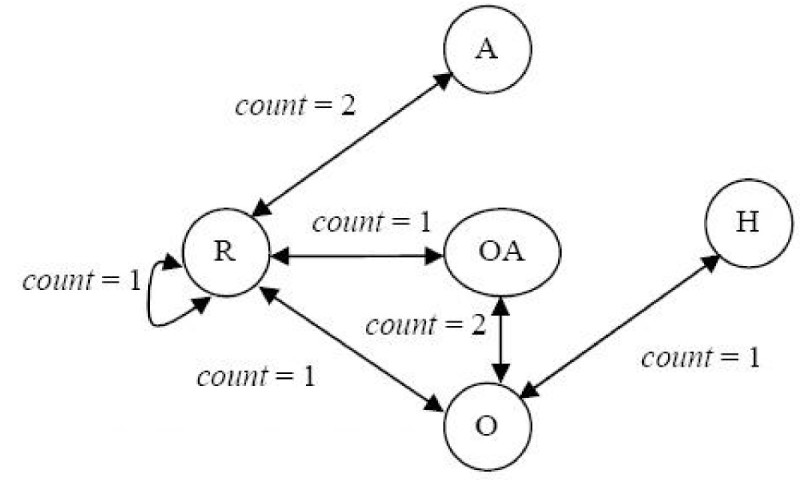
**The De Bruijn graph** ***D*** **for the VSMMD shown in Figure 9**. Note: We use 'O' to denote O#O#O#O#O#O and 'OA' to denote O#O#O#O#A.

Although we have been referencing the template in describing the construction of *D*, it should be clear that it is possible to accomplish the generation of *D* by processing the descriptor that represents this template. Figure 11 illustrates the De Bruijn graph *D* generated from the VSMMD shown in Figure 9.

**Figure 12:**
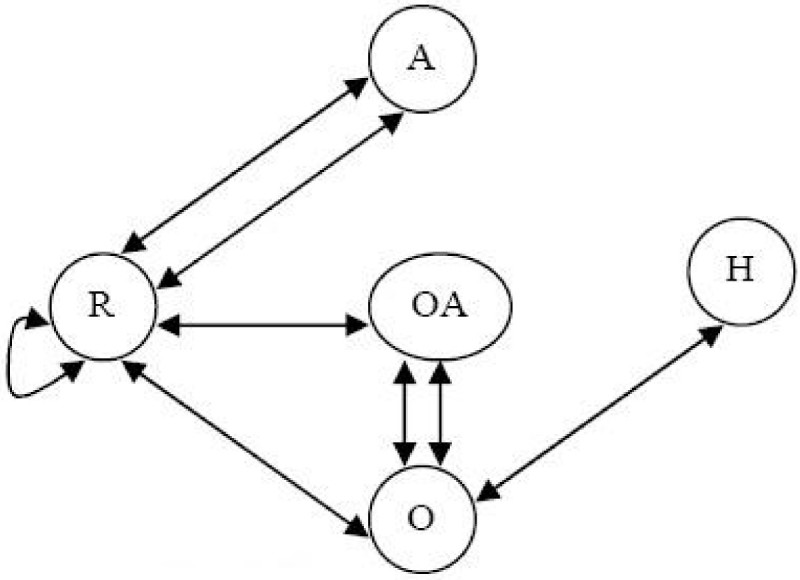
**The Expanded graph** ***M***. Note: We use 'O' to denote O#O#O#O#O#O and 'OA' to denote O#O#O#O#A.

The De Bruijn graph can be *expanded* by replacing each edge carrying weight *c* with *c* unweighted edges, each with the same direction as the original edge. Figure 12 illustrates this expansion. Let *M* be the resulting unweighted De Bruijn graph.

From the VSMMD, we know the exact number of vertices that should appear in the chemical structure template. With this information, we can derive a chemical structure template from the De Bruijn graph by finding an Euler circuit of *M*.

#### All possible Euler circuits

An Euler circuit is a circuit on the graph such that each edge is traversed exactly once. Each traversal of an edge corresponds to the consumption of one instance of a component in the VSMMD. The problem of finding an Eulerian circuit of a graph is well known and there exists a linear time algorithm for its derivation [37]. The following is the pseudo-code of the Euler circuit algorithm.

EULER (*q*)

1. Path ← none
2. For each unmarked edge *e* leaving *q* do
3.          Mark(*e*)
4.          Path A ← EULER(*opposite vertex*(*e*)) || Path
5. Return Path

**Figure 13:**
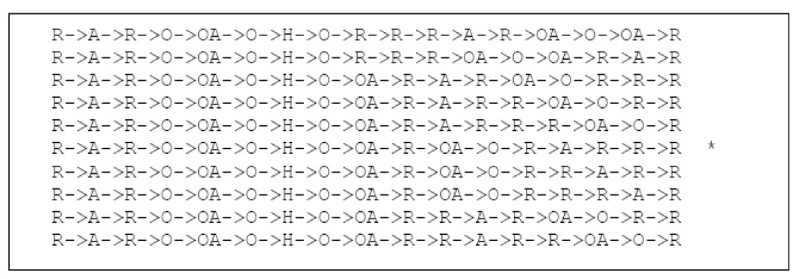
**Some possible Euler circuits**.

Each Euler circuit will represent the extraction of a unique chemical structure template from the De Bruijn Graph *M*. Figure 13 shows a subset of all the Euler circuits that can be generated. The circuit labelled with a '*' corresponds to the chemical structure template illustrated in Figure 10. The total number of possible Euler circuits for the chemical structure in Figure 9 is 2700.

In order to generate all possible chemical structures associated with the VSMMD, we have to find all Euler circuits. Different chemical structure templates correspond to the different possible orderings when traversing outgoing edges of each vertex. This produces a factorial explosion with respect to the number of outgoing edges of each vertex. Thus, finding all Euler circuits is not feasible.

#### Probabilistic Euler paths

To overcome this, we have developed an algorithm that generates Eulerian circuits by doing a guided walk of the graph. During the walk we choose an outgoing edge in a probabilistic fashion. The choice is dependent on statistics that are gathered from the descriptors in the training set. To accomplish this, we have built a statistical model that is used to estimate the probability of an Euler circuit.

Let *E* denote a path of *N* edges, that is, *E* = *e*_1_, *e*_2_,...,*e*_*N*_. Then, by the probability chain rule, we can obtain:
33

To estimate the conditional probabilities *P* (*e*_*i*_|*e*_1_, *e*_2_,⋯,*e*_*i*-1_), we need training data consisting of a large number of Euler circuits each corresponding to some particular molecular template. One can obtain these conditional probability distributions from the training data by keeping statistics on the dependency between the next edge to traverse and the history of the previously traversed edges. Seen as probabilities of traversal, we of course use normalized values so that the probabilities of all possible "next-edges" sum to 1.0.

To simplify the statistical model, independence assumptions are made so that each edge depends only on the last *t* edges. Consequently, we have a Markov model that provides an approximation of how the fragments, each labelled with physicochemical properties, are connected within the template. More precisely, our model predicts traversal of *ei* based on previously traversed edges *e*_*i*-1_, *e*_*i*-2_,⋯,*e*_*i*-*t*_. Formally, this is described as:
34

If we could handle unlimited amounts of training data, the maximum likelihood estimate of *P* (*e*_*i*_|*e*_*i*-*t*+1_,⋯,*e*_*i*-1_) would be:
35

where *c*(*e*_*i*-*t*_,⋯,*e*_*i*-1_, *e*_*i*_) is the number of times the edge sequence *e*_*i*-*t*_,⋯,*e*_*i*-1_, *e*_*i*_is seen in the training data.

**Figure 14:**
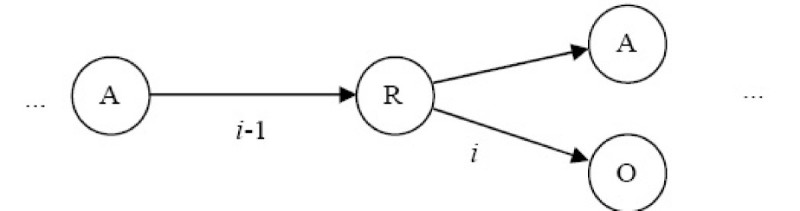
**An example in edges traversal**.

As an example, consider Figure 14 with *t* = 1. In order to determine the edge to be traversed next when at the node labelled "R", we consult the associated probabilities: *P*(R - O | A - R) and *P*(R - A | A - R). We traverse the edge with the largest probability first.

A threshold *h* can also be used as a cut-off to limit the number of edges that the algorithm should examine in an effort to sidestep the factorial explosion that can occur without this limitation. With this understanding, we can compute the overall probability of each Euler circuit using equation (34).

**Figure 15:**
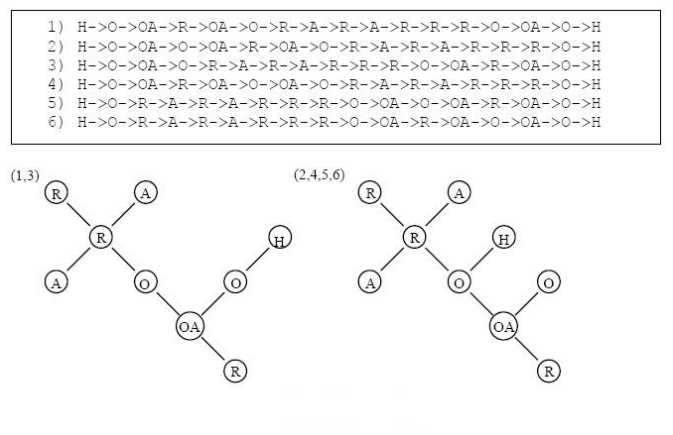
**Six highest probability Euler Circuits for VSMMD shown in Figure 9 and the corresponding chemical structure templates**. Note: We use 'O' to denote O#O#O#O#O#O and 'OA' to denote O#O#O#O#A.

With *t* = 1 and *h* = 2, a total of 102 Euler circuits are generated. The six Euler circuits with highest probability are shown in Figure 15. They corresponded to two unique chemical templates. Templates 1 and 3 are the exact templates derived from the chemical structure shown in Figure 10.

There are several related research papers that attempt to retrieve the order of elements that are part of a larger structure using Eulerian circuits. Cortes *et al*. [38] retrieve the order of words in documents using an Eulerian circuit approach. Pevzner *et al*. [39] assembly DNA fragments using an Eulerian circuit.

As mentioned in [29], in general, there was no exact pre-image in the input space; The pre-image returned by the algorithm was an approximation and so it was compromised by approximation errors. Because of these approximation errors, the following problems may exist:

- The pre-image vector may consist of non-integer components.
- The pre-image vector may not form a fully connected De Bruijn Graph.

Our solution to overcome the first problem is to round the components to obtain integer counts. To deal with the case where the graph is not connected, the all-possible Euler circuits algorithm is called at each vertex whose outgoing edges are not all marked. The resulting path is the concatenation of the paths returned by different calls to the all-possible Euler algorithm. More precisely, a bidirectional edge with the largest conditional probability based on previously traversed edges in the path, (using the same Markov model that we set up in the previous subsection), is added to connect two Euler paths together.

**Figure 16:**
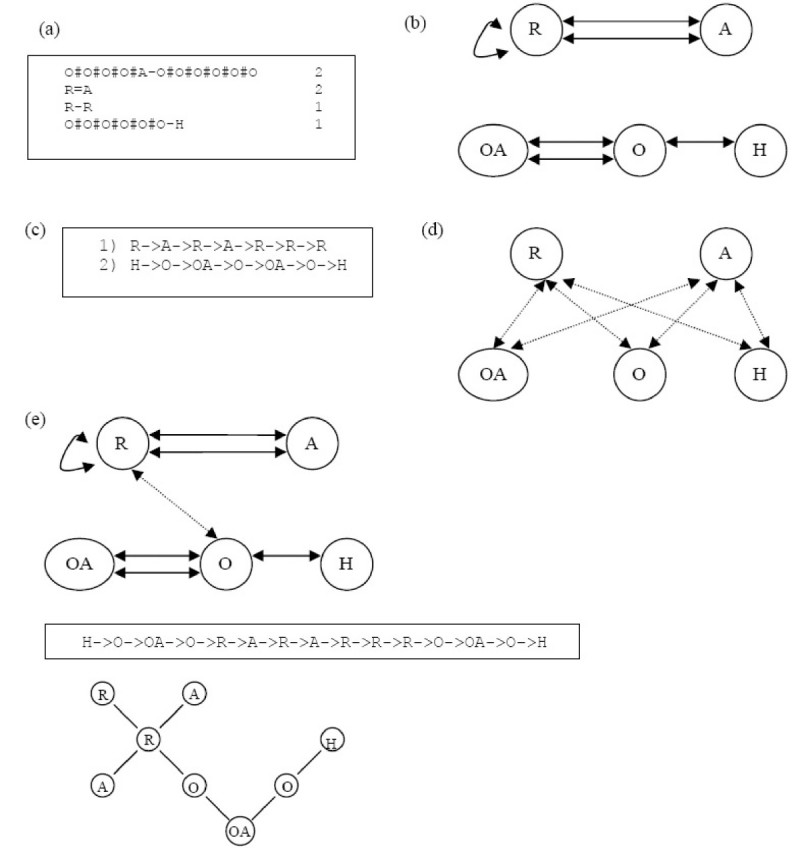
**A case where the pre-image vector did not form a fully connected De Bruijn Graph**.

Consider the pre-image example given in Figure 16(a), the corresponding expanded De Bruijn Graph is given in Figure 16(b). The all-possible Euler algorithm is called at each vertex whose outgoing edges are not all marked. The highest probability Euler circuits for the disjoint De Bruijn Graph are given in Figure 16(c). To determine the concatenation location of the two Euler circuits, our algorithm considers all the possible connections between the two disjoint parts of the graph. All the possible connections are illustrated in Figure 16(d). These possible connections are evaluated using the same Markov model that we set up before to calculate the Euler circuits. Among all the possible connections, the P(R-O|R-R) value gives the highest probability. Thus a bi-directional edge between node 'R' and node 'O', is added to connect the two disjointed parts together. The final De Bruijn Graph, the concatenated Euler circuit and the corresponding graph template are shown in Figure 16(e).

## Results

### Data

In our previous work [24], eight different data sets were used to test the ability of the VSMMD to predict biological activities. All these data sets contain real valued QSAR inhibitor data. The eight QSAR data sets are from Sutherland *et al*. [27]. We chose one data set from these eight data sets to demonstrate the effectiveness of the recovery algorithm when applied to our VSMMD.

**Figure 17:**
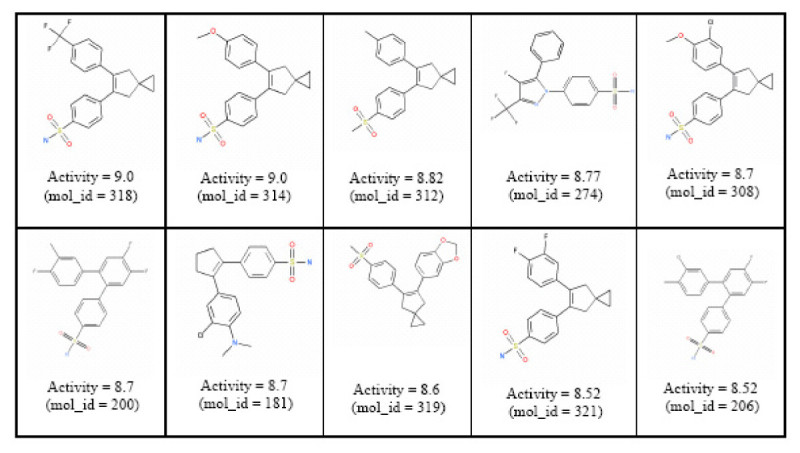
**Ten highest active compounds in the COX-2 training set**.

The data set we chose contains 322 cyclooxygenase-2 (COX2) inhibitors collected by Seibert and colleagues [40] and subsequently utilized in a QSAR study by Chavatte *et al*. [41] with each inhibitor having pIC_50_ values ranging from 5.5 to 8.9. We chose this data set because training samples in the COX2 data set were presented using diagrams of molecular structures. This allowed us to compare our generated chemical structure templates with the given molecules in the data set. It should also be noted that, despite the similarity of molecules displayed in Figure 17, the entire set of 322 COX2 inhibitors is spread over 20 different scaffolds and so the training set has a reasonable structural diversity.

The same inverse-QSAR procedure was applied to the remaining seven data sets; the closest matching molecule in the test set for the generated chemical template is shown in the last subsection.

In our experiments, the data were separated into the same training and testing sets as specified by Sutherland *et al*. [27].

### Implementation Details

To identify the physicochemical properties of each atom, we implemented our descriptor generation program with help from the chemical development kit (CDK) [42, 43] programmed in Java. As illustrated in Figure 3, descriptors were calculated and the kernel matrix *K* was generated in a few seconds for each complete data set. For the pre-image algorithm and the feature space algorithm, we used MATLAB to perform the required calculations. For the recovery phase, we implemented the Probabilistic Euler Paths algorithm in Java.

### Verification of the Inverse Mapping – Test Result

**Figure 18:**
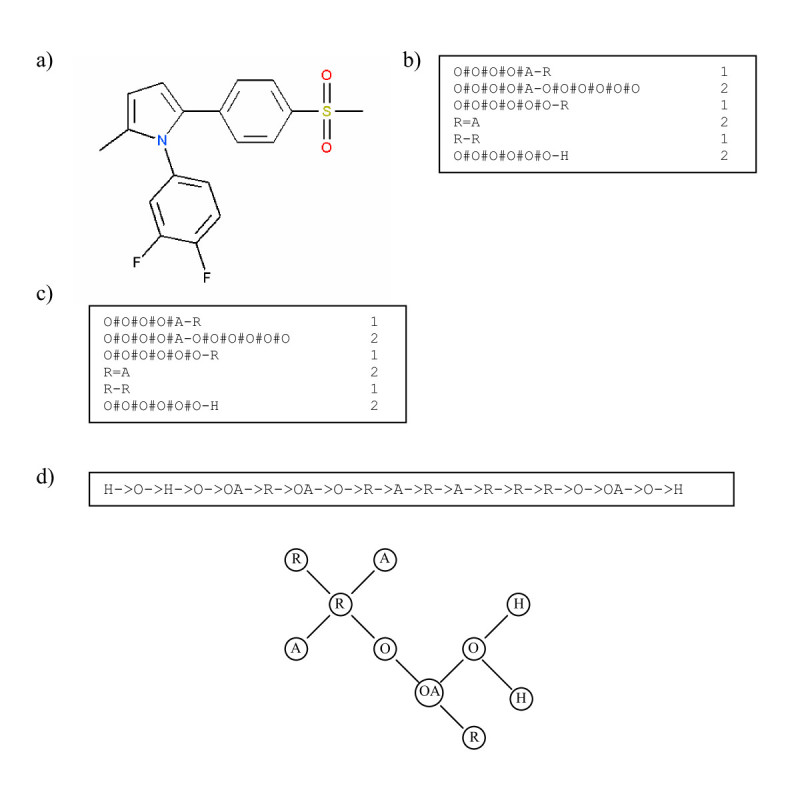
**Verification test result**.

To verify our proposed inverse approach, we picked one molecule in the COX2 training set randomly as shown in Figure 18(a). We then generated the VSMMD for each of the compounds in the training set. The corresponding VSMMD for the chosen molecule is shown in Figure 18(b). Next, we implicitly mapped this VSMMD to the kernel feature space using a Gaussian kernel function as stated in equation (5). Instead of generating a new point in the feature space, we used the pre-image approximation algorithm to compute the pre-image of this feature space point. The corresponding pre-image is shown in Figure 18(c). Finally, we applied our VSMMD recovery algorithm to obtain a chemical template. With *t* = 1 and *h* = 3, we generated the Euler circuit with the highest probability and the corresponding chemical structure templates are shown in Figure 18(d). From this result, we observed that our approach is able to generate a chemical template corresponding to the original chosen molecule.

### Inverse-QSAR Test Results

Recall that our inverse-QSAR approach contains five steps. The first two steps are to perform QSAR analysis. In the first step, we generated the VSMMD for the compounds in the training set. Then, in the second step, we implicitly mapped the VSMMD to the kernel feature space using an appropriate kernel function for classification. The results of the forward QSAR can be found in our previous paper [24].

The third step was to design or to generate a new point in the kernel feature space using a kernel feature space algorithm. To demonstrate our approach, we formed a new point in the feature space by using the ten highest active compounds in the training set. The center of the minimum enclosing and maximum excluding hypersphere was obtained using equation (19). Figure 17 shows these ten compounds.

**Figure 19:**
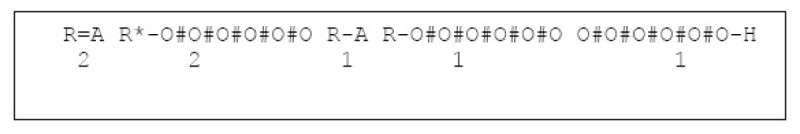
**The pre-image VSMMD of the center of the minimum enclosing and maximum excluding hyperspheres**.

In the fourth step, we mapped the feature space point back into the input space using the pre-image approximation algorithm. In this case, we used the Kwok and Tsang algorithm [29] described in the pre-image problem subsection to map the center of the minimum enclosing and maximum excluding hypersphere back into the input space. Figure 19 illustrates the derived VSMMD.

**Figure 20:**
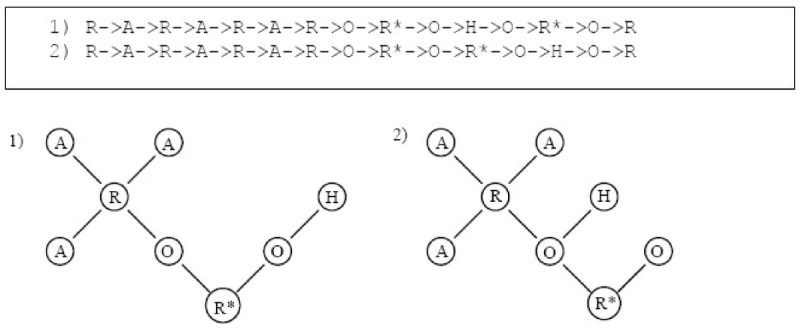
**Two Euler circuits with the highest probability for the pre-image VSMMD in Figure 19 and the corresponding chemical structure templates**. Note: We use 'O' to denote O#O#O#O#O#O and 'R*' to denote the cyclopentene ring.

The last step concerned building the molecular structure template using our VSMMD recovery algorithm described in the recovery subsection. Since the center of the minimum enclosing and maximum excluding hypersphere was derived from the ten highest active compounds in the training set, we assumed that the new derived compounds should look similar to these ten compounds. With this assumption the path probability was calculated. Setting *t* = 1 and *h* = 3, we generated two Euler circuits and the corresponding chemical structure template is shown in Figure 20.

### Notes on Test Results

In order to investigate whether the pre-image VSMMD is reasonable, we performed a more detailed analysis of the COX-2 data set. In the pre-image VSMMD as shown in Figure 20, the cyclopentene ring can be found in one of the descriptors. From medicinal chemistry studies, we know that cyclopentene derivatives are one of the first series of diaryl-substituted cycles that have been well known as COX-2 inhibitors [44, 45]. This empirical evidence demonstrates that our generated pre-image VSMMD is able to capture important properties of the ten most active molecules.

When we performed a high throughput screening on the test set using the generated chemical structure template, the following molecule was identified as an exact match to the template.

**Figure 21:**
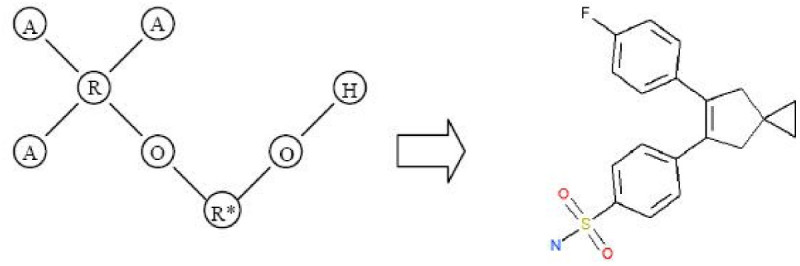
**Matching molecule in the test set**. Note: We use 'O' to denote O#O#O#O#O#O and 'R*' to denote the cyclopentene ring.

The molecule in Figure 21 has a pIC50 value of 8.52, and it was one of the highest active molecules in the testing set. From this result, we demonstrated that our strategy was able to generate a high affinity molecule using only data in the training set. We were able to claim that the generated molecule was a high affinity molecule because it appeared as such in the testing set. In practice, the success of the algorithm would have to be assessed by using a wet lab procedure to determine the affinity of the generated molecule.

**Figure 22:**
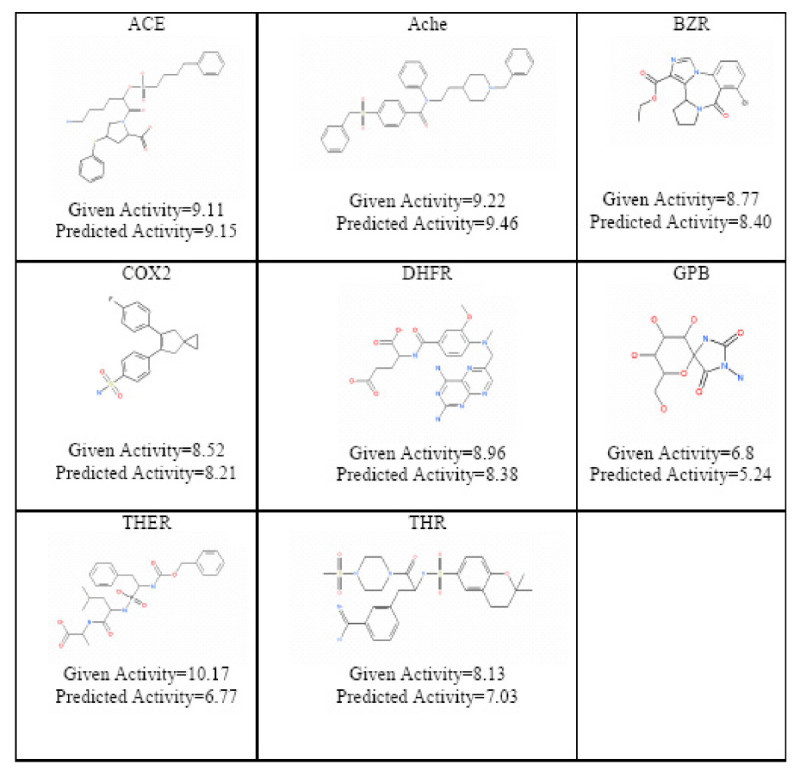
**The closest matching molecule in the test set for the generated chemical template across 8 data sets**.

The inverse-QSAR procedure was applied to all eight data sets; the closest matching molecule in the test set for the generated chemical template across 8 data sets is shown in Figure 22. A quantitative evaluation of the generated molecules was performed by implicitly mapping the VSMMD of each molecule to the kernel feature space for regression analysis. The regression results are also shown in Figure 22.

## Discussion

In kernel-based learning, the usual assumption is that the data pairs , in the training set, come from a source that provides these samples in an independent identically distributed (i.i.d.) fashion according to an unknown probability distribution *P*(*x*, *y*). Furthermore, the test examples are assumed to come from the *same* distribution [46]. In an ideal situation, the collection of molecular descriptors in the training set follow a probability distribution that is only determined by the interactions between ligands and the binding site. In practise this does not happen. The selection of members in the training set may involve a significant amount of bias due to human involvement in its creation:

- Selection of members of the training set may be restricted by rules that exclude molecules that are not "drug like".
- Since the training set involves molecules that have been assessed for binding affinity, they had to be synthesized and may be part of a suite of molecules for which the synthesis was not overly complicated.
- Furthermore, the molecular descriptors in the training set may show various types of repetition, (for example, the repeated occurrence of some type of scaffold). This may or may not be intended.

As a consequence of these issues, the learning algorithm will produce a predictor that is taking into account both a biological process and the human activity intrinsic to the formation of the training set. More significantly, there is the demand that future test molecules come from the same probability distribution. Statistical learning theory will guarantee certain generalization bounds, but only if these demands are met. In effect, the theory tells us that if test samples come from a source, such as a virtual screening library that is not characterized by the same rules of formation as the training set – then all bets are off.

In the constructive approach that has been described in this paper, it is clear that we are also limited by the information that is intrinsic to a training set. But beyond this, the strategy significantly differs from virtual screening. Instead of trying to find a new molecule in a database that should exhibit the same *P*(*x,y*) characteristics, we side step this requirement (which may be difficult to guarantee) and we build a new drug candidate using only the information that is strictly contained in the training set itself.

## Conclusion

While molecular fragments have been used in research studies for dealing with quantitative structure-activity relationship problems, we have further evolved this strategy to include a reverse engineering mechanism.

These mechanisms include:

1. the use of a kernel feature space algorithm to design or modify descriptor image points in a feature space
2. the deployment of a pre-image algorithm to map the descriptor image points in the feature space back to the input space of the descriptors, and
3. the design of a probabilistic strategy to convert new descriptors into meaningful chemical graph templates

As reported in earlier papers, our modeling has produced very effective algorithms to predict drug-binding affinities and to predict multiple binding modes [24]. We have now extended our modeling approach to the development of algorithms that derive new descriptors and then facilitate the reverse engineering of such a descriptor. This is a very desirable capability for a molecular descriptor [3].

The most important aspect of our research is the presentation of strategies that actually generate the structure of a new drug candidate. This is substantially different from methodologies that depend on database screening to get new drug candidates. While our approach can support such an endeavour, it is not our primary goal. In fact, we are quite concerned that database screening, done using a predictor derived from a statistical learning algorithm, is subject to procedural demands that may be difficult to maintain. We are referring to statistical learning theory that guarantees the success of a predictor, but only when the test sample is drawn from a data source that has the same probability distribution as that characterizing the training set.

In the applications of statistical learning to database screening, the predictor may be applied to test molecules that have very little relationship to the training data. In these cases, the predictor is optimistically treated as if it actually incorporates an algorithm that has some firm and direct relationship to the biological context of the problem. As mentioned by Good *et al*. [47], the conclusion of a QSAR analysis can be profoundly altered by how the test set was derived. Traditionally, this concern was usually addressed through the design of complicated and time consuming validation experiments [48] to ensure that the predictor will not generate a misleading conclusion. In our approach, we have avoided such concerns. While the training set is still used to generate a new image point in the feature space, the reverse engineering just described allows us to develop a template for a new drug candidate that is independent of issues related to probability distribution constraints placed on test set molecules.

## Authors’ original submitted files for images

Below are the links to the authors’ original submitted files for images. Authors’ original file for figure 1 Authors’ original file for figure 2 Authors’ original file for figure 3 Authors’ original file for figure 4 Authors’ original file for figure 5 Authors’ original file for figure 6 Authors’ original file for figure 7 Authors’ original file for figure 8 Authors’ original file for figure 9 Authors’ original file for figure 10 Authors’ original file for figure 11 Authors’ original file for figure 12 Authors’ original file for figure 13 Authors’ original file for figure 14 Authors’ original file for figure 15 Authors’ original file for figure 16 Authors’ original file for figure 17 Authors’ original file for figure 18 Authors’ original file for figure 19 Authors’ original file for figure 20 Authors’ original file for figure 21 Authors’ original file for figure 22
