# Supplementary figures and images for: A constructive approach for discovering new drug leads: Using a kernel methodology for the inverse-QSAR problem
Source: J Cheminform. 2009 Apr 28;1:4. doi: 10.1186/1758-2946-1-4 (PMC2816860; doi:10.1186/1758-2946-1-4)

*Chemical space*

*Input space of  
descriptors*

*Feature space*

*VSMMD*

$\phi$

*recovery*

$\phi^{-1}$

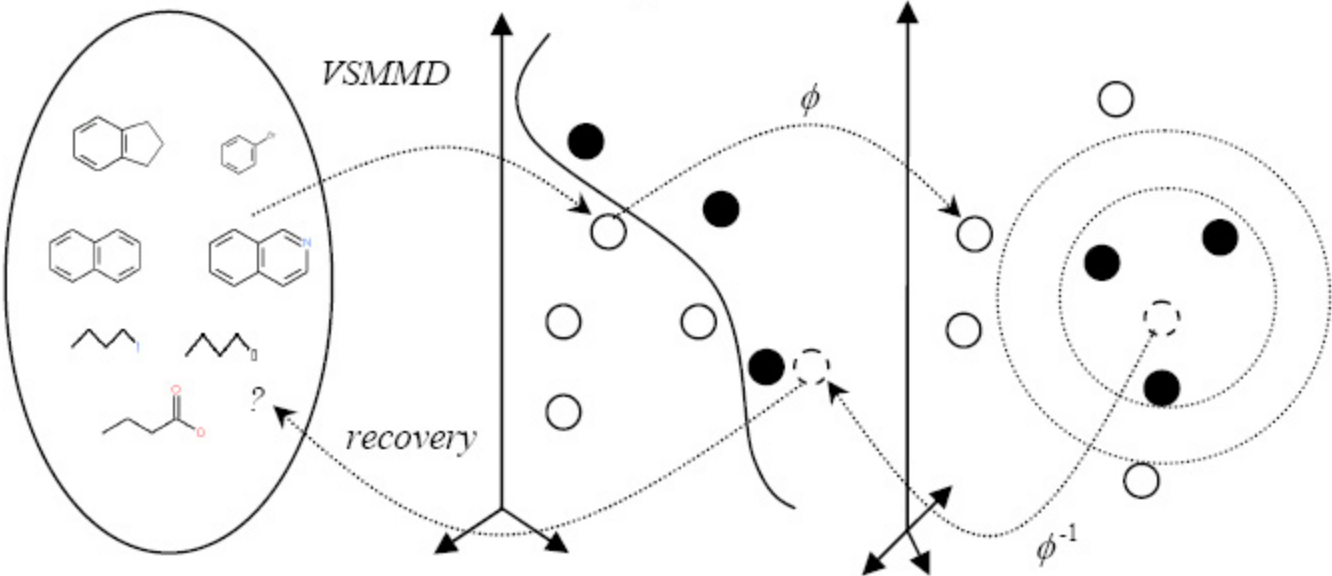

Supplement: Supplementary file 1 — Authors’ original file for figure 1 [file 13321_2009_4_MOESM1_ESM.pdf]

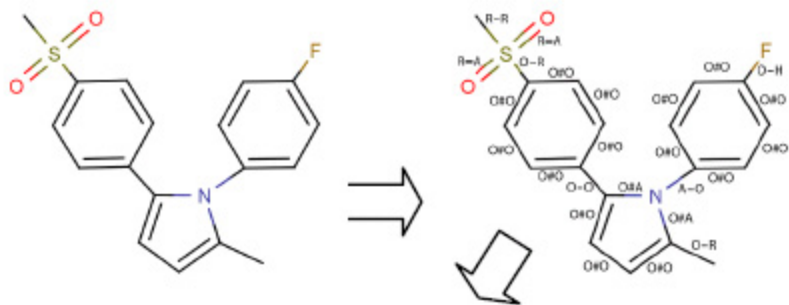

R-R R=A R=A O-R O#O O#O O#O O#O O#O O#O O-O  
 O#A O#A O-R O#O O#O O#O A-O O#O O#O O#O O#O  
 O#O O#O O-H

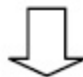

R-R R=A O-R O#O O-O O#A A-O O-H  
 1 2 2 15 1 2 1 1

Supplement: Supplementary file 3 — Authors’ original file for figure 3 [file 13321_2009_4_MOESM3_ESM.pdf]

*Input Space*

*Feature Space*

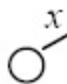

$x$

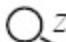

$z$

$\phi$

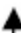

$\phi$

$\phi(x)$

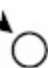

$\phi(z)$

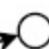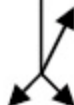

Supplement: Supplementary file 4 — Authors’ original file for figure 4 [file 13321_2009_4_MOESM4_ESM.pdf]

*Chemical space*

*Input space of  
descriptors*

*Feature space*

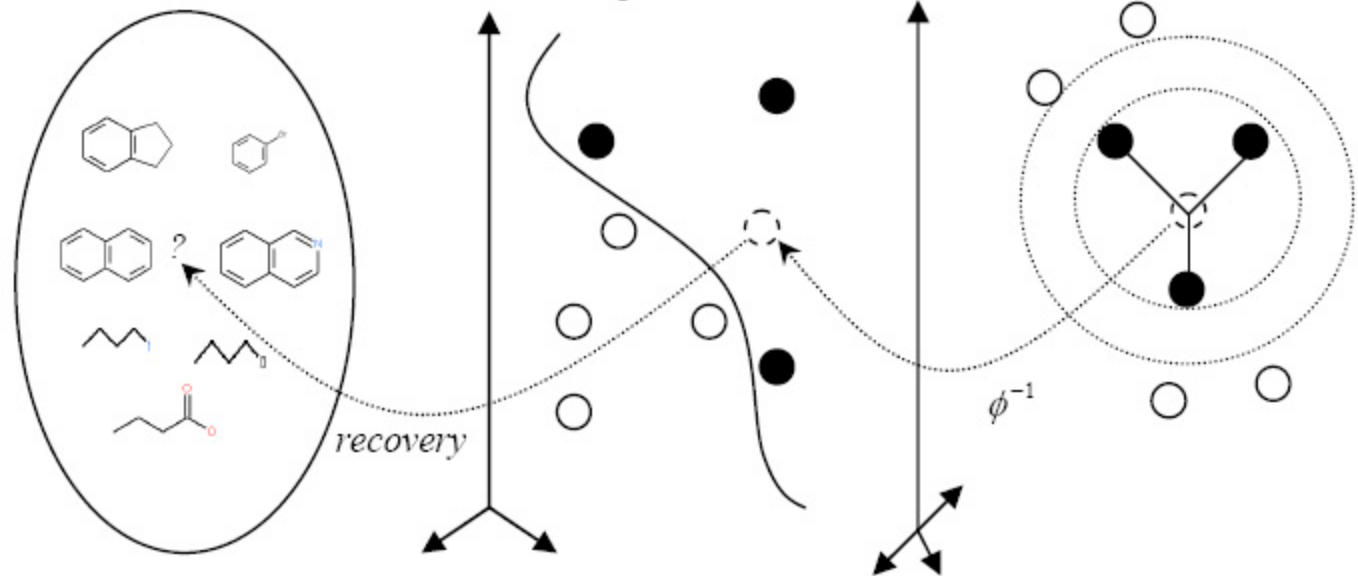

Supplement: Supplementary file 5 — Authors’ original file for figure 5 [file 13321_2009_4_MOESM5_ESM.pdf]

*Feature space*

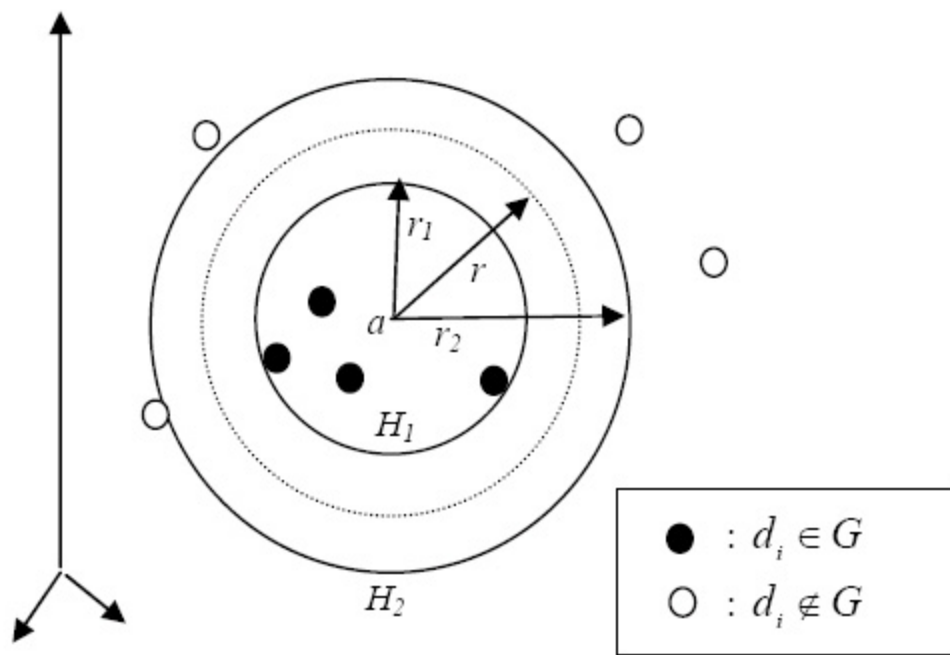

Supplement: Supplementary file 6 — Authors’ original file for figure 6 [file 13321_2009_4_MOESM6_ESM.pdf]

*Input space of  
descriptors*

$\phi$

*Feature space*

$d^*$

$\psi$

$\phi^{-1}$

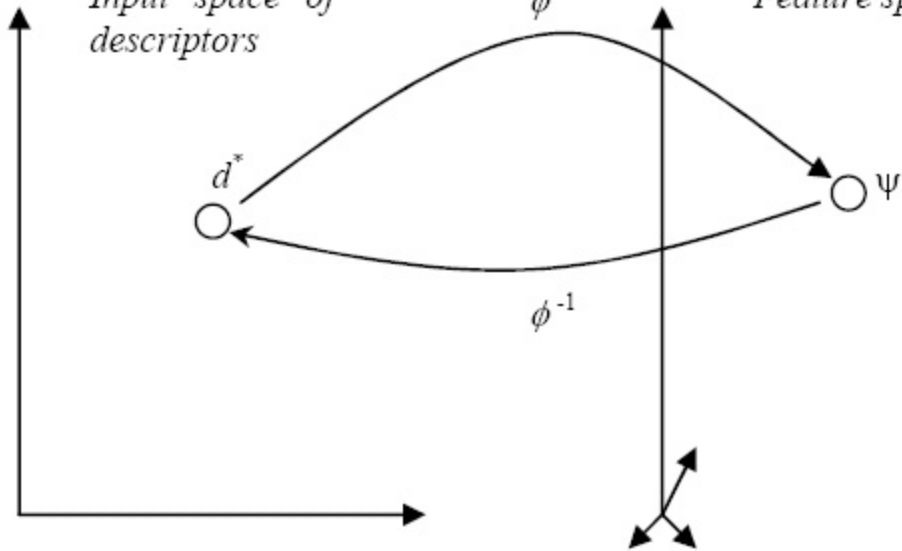

Supplement: Supplementary file 7 — Authors’ original file for figure 7 [file 13321_2009_4_MOESM7_ESM.pdf]

*Input Space*

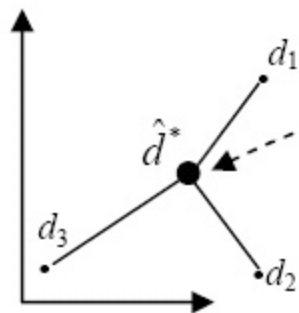

*Feature Space*

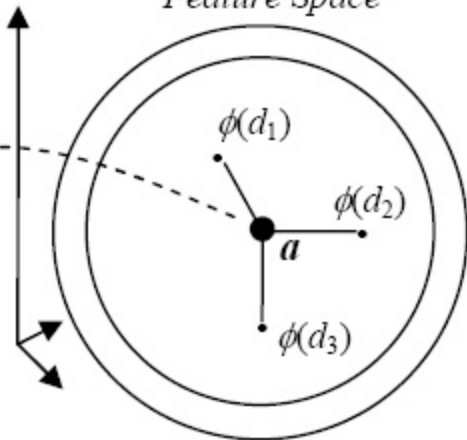

$\hat{d}^*$

$d_1$

$d_2$

$d_3$

$\phi(d_1)$

$\phi(d_2)$

$\phi(d_3)$

$a$

Supplement: Supplementary file 8 — Authors’ original file for figure 8 [file 13321_2009_4_MOESM8_ESM.pdf]

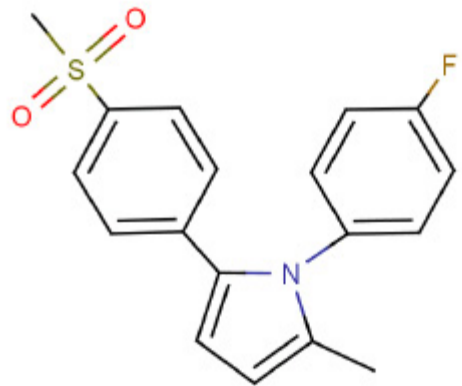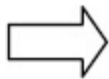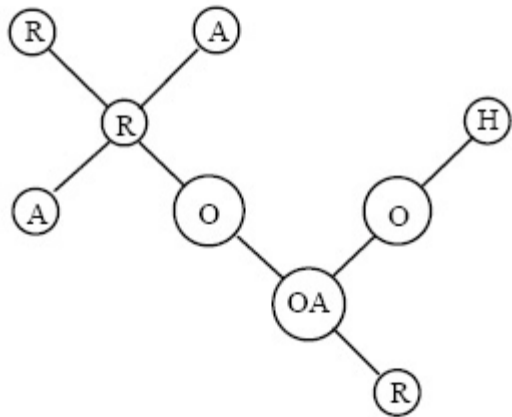

Supplement: Supplementary file 10 — Authors’ original file for figure 10 [file 13321_2009_4_MOESM10_ESM.pdf]

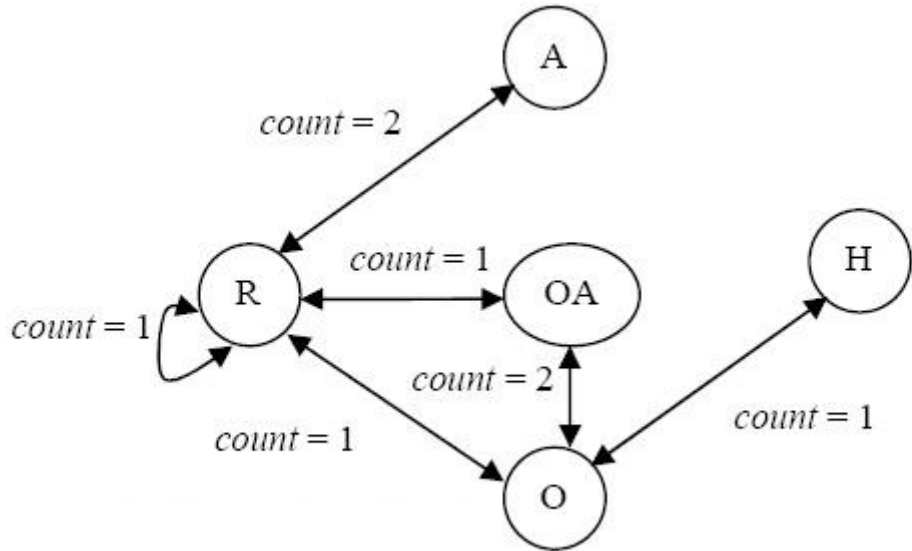

Supplement: Supplementary file 11 — Authors’ original file for figure 11 [file 13321_2009_4_MOESM11_ESM.pdf]

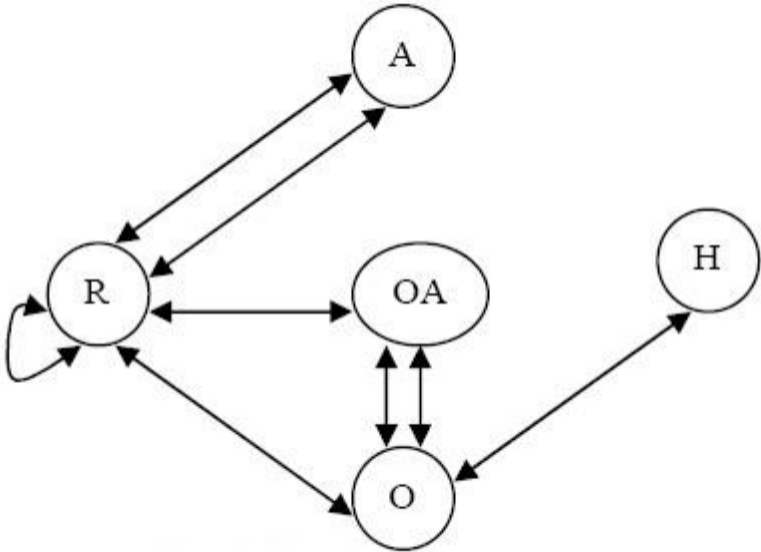

Supplement: Supplementary file 12 — Authors’ original file for figure 12 [file 13321_2009_4_MOESM12_ESM.pdf]

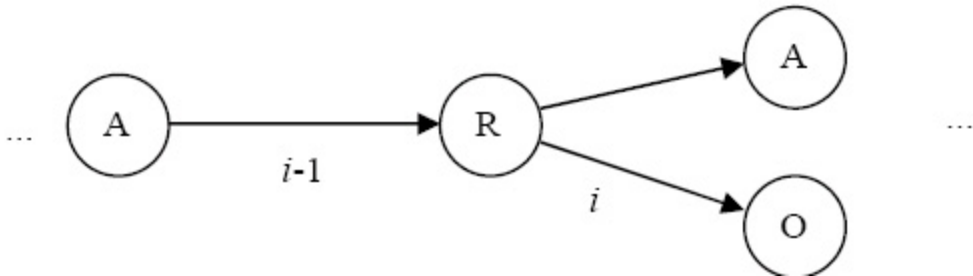

Supplement: Supplementary file 14 — Authors’ original file for figure 14 [file 13321_2009_4_MOESM14_ESM.pdf]

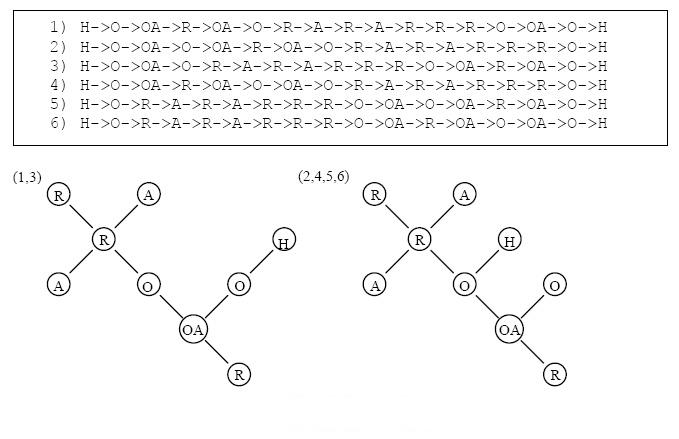

Supplement: Supplementary file 15 — Authors’ original file for figure 15 [file 13321_2009_4_MOESM15_ESM.jpeg]

(a)

|                       |   |
|-----------------------|---|
| O#O#O#O#A-O#O#O#O#O#O | 2 |
| R=A                   | 2 |
| R-R                   | 1 |
| O#O#O#O#O#O-H         | 1 |

(b)

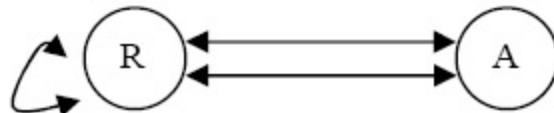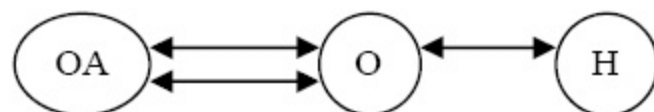

(c)

1) R->A->R->A->R->R->R  
 2) H->O->OA->O->OA->O->H

(d)

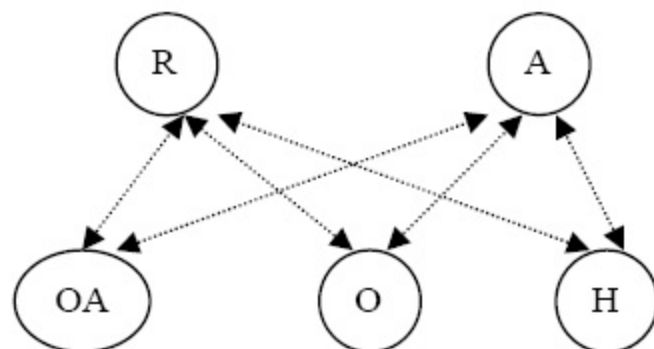

(e)

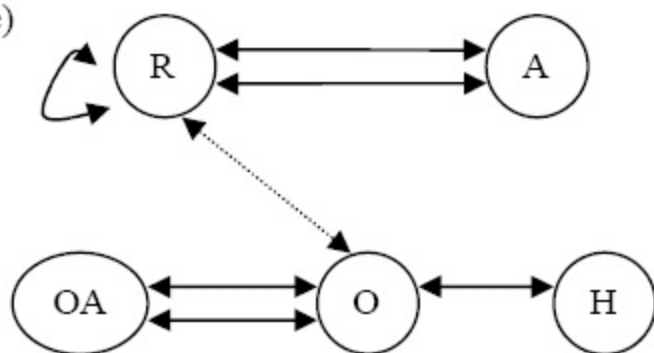

H->O->OA->O->R->A->R->A->R->R->R->O->OA->O->H

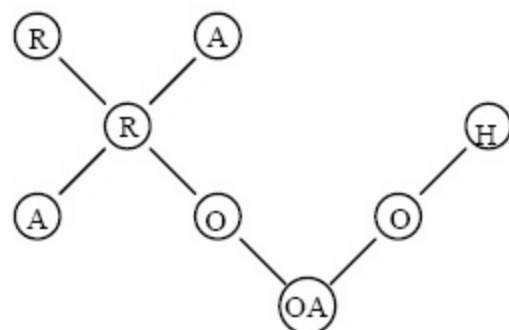

Supplement: Supplementary file 16 — Authors’ original file for figure 16 [file 13321_2009_4_MOESM16_ESM.pdf]

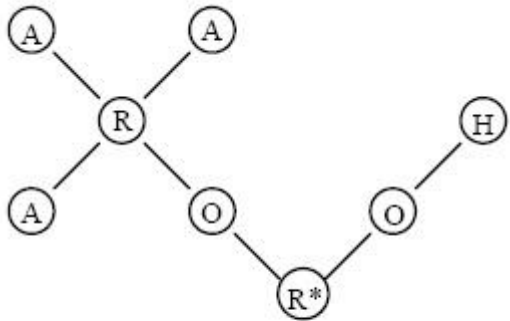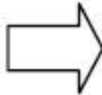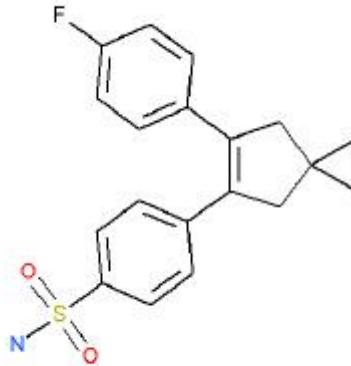

Supplement: Supplementary file 21 — Authors’ original file for figure 21 [file 13321_2009_4_MOESM21_ESM.pdf]
