# Supplementary material for: A constructive approach for discovering new drug leads: Using a kernel methodology for the inverse-QSAR problem
Source: J Cheminform. 2009 Apr 28;1:4. doi: 10.1186/1758-2946-1-4 (PMC2816860; doi:10.1186/1758-2946-1-4)
Supplement: Supplementary file 2 — Authors’ original file for figure 2 [file 13321_2009_4_MOESM2_ESM.pdf]

hydrogen bond acceptor	A	Atom Type (A_T)
hydrogen bond donor	D	
positively charged	P	
negatively charged	N	
aromatic rings	O	
halogen atom	H	
none of the above	R	
single bond	–	Bond Type (B_T)
double bond	=	
Aromatic bond	#	
