# Supplementary material for: A constructive approach for discovering new drug leads: Using a kernel methodology for the inverse-QSAR problem
Source: J Cheminform. 2009 Apr 28;1:4. doi: 10.1186/1758-2946-1-4 (PMC2816860; doi:10.1186/1758-2946-1-4)
Supplement: Supplementary file 9 — Authors’ original file for figure 9 [file 13321_2009_4_MOESM9_ESM.pdf]

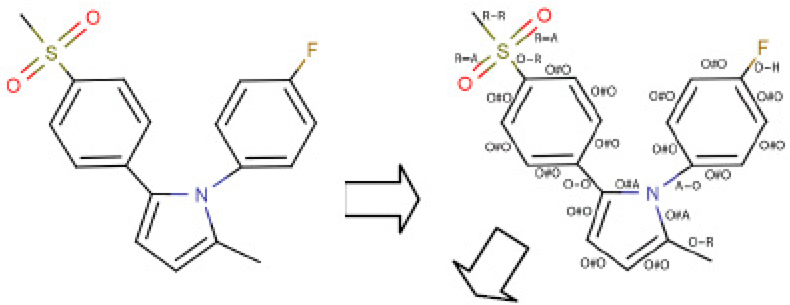

O#O#O#O#A-R O#O#O#O#A-O#O#O#O#O#O
O#O#O#O#A-O#O#O#O#O#O O#O#O#O#O#O-R
R=A R=A R-R O#O#O#O#O#O-H

O#O#O#O#A-R	1
O#O#O#O#A-O#O#O#O#O#O	2
O#O#O#O#O#O-R	1
R=A	2
R-R	1
O#O#O#O#O#O-H	1
