# Supplementary material for: A constructive approach for discovering new drug leads: Using a kernel methodology for the inverse-QSAR problem
Source: J Cheminform. 2009 Apr 28;1:4. doi: 10.1186/1758-2946-1-4 (PMC2816860; doi:10.1186/1758-2946-1-4)
Supplement: Supplementary file 13 — Authors’ original file for figure 13 [file 13321_2009_4_MOESM13_ESM.pdf]

```
R->A->R->O->OA->O->H->O->R->R->R->A->R->OA->O->OA->R
R->A->R->O->OA->O->H->O->R->R->R->OA->O->OA->R->A->R
R->A->R->O->OA->O->H->O->OA->R->A->R->OA->O->R->R->R
R->A->R->O->OA->O->H->O->OA->R->A->R->R->OA->O->R->R
R->A->R->O->OA->O->H->O->OA->R->A->R->R->R->OA->O->R
R->A->R->O->OA->O->H->O->OA->R->OA->O->R->A->R->R->R   *
R->A->R->O->OA->O->H->O->OA->R->OA->O->R->R->A->R->R
R->A->R->O->OA->O->H->O->OA->R->OA->O->R->R->R->A->R
R->A->R->O->OA->O->H->O->OA->R->R->A->R->OA->O->R->R
R->A->R->O->OA->O->H->O->OA->R->R->A->R->R->OA->O->R
```
