# Supplementary material for: A constructive approach for discovering new drug leads: Using a kernel methodology for the inverse-QSAR problem
Source: J Cheminform. 2009 Apr 28;1:4. doi: 10.1186/1758-2946-1-4 (PMC2816860; doi:10.1186/1758-2946-1-4)
Supplement: Supplementary file 17 — Authors’ original file for figure 17 [file 13321_2009_4_MOESM17_ESM.pdf]

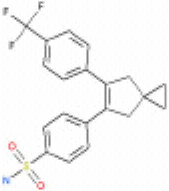<br>Activity = 9.0<br>(mol_id = 318)	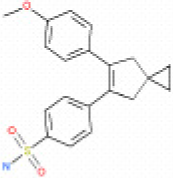<br>Activity = 9.0<br>(mol_id = 314)	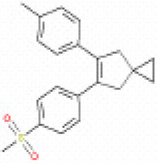<br>Activity = 8.82<br>(mol_id = 312)	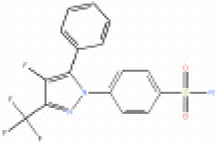<br>Activity = 8.77<br>(mol_id = 274)	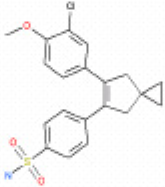<br>Activity = 8.7<br>(mol_id = 308)
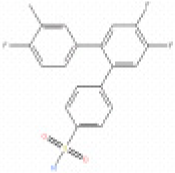<br>Activity = 8.7<br>(mol_id = 200)	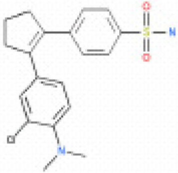<br>Activity = 8.7<br>(mol_id = 181)	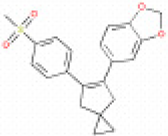<br>Activity = 8.6<br>(mol_id = 319)	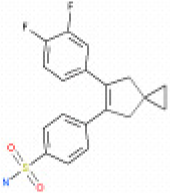<br>Activity = 8.52<br>(mol_id = 321)	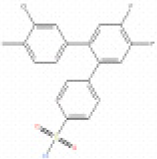<br>Activity = 8.52<br>(mol_id = 206)
