# Supplementary material for: A constructive approach for discovering new drug leads: Using a kernel methodology for the inverse-QSAR problem
Source: J Cheminform. 2009 Apr 28;1:4. doi: 10.1186/1758-2946-1-4 (PMC2816860; doi:10.1186/1758-2946-1-4)

a)

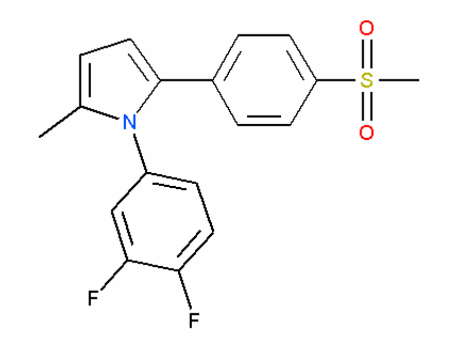

b)

```
O#O#O#O#A-R                 1
O#O#O#O#A-O#O#O#O#O#O       2
O#O#O#O#O#O-R               1
R=A                         2
R-R                         1
O#O#O#O#O#O-H               2
```

c)


```
O#O#O#O#A-R                 1
O#O#O#O#A-O#O#O#O#O#O       2
O#O#O#O#O#O-R               1
R=A                         2
R-R                         1
O#O#O#O#O#O-H               2
```


d)

```
H->O->H->O->OA->R->OA->O->R->A->R->A->R->R->R->O->OA->O->H
```

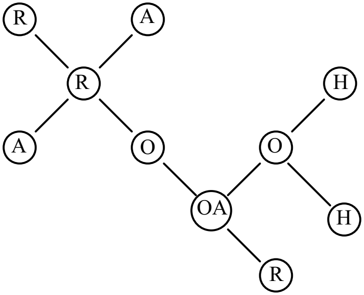

Supplement: Supplementary file 18 — Authors’ original file for figure 18 [file 13321_2009_4_MOESM18_ESM.pdf]
