# Supplementary material for: A constructive approach for discovering new drug leads: Using a kernel methodology for the inverse-QSAR problem
Source: J Cheminform. 2009 Apr 28;1:4. doi: 10.1186/1758-2946-1-4 (PMC2816860; doi:10.1186/1758-2946-1-4)
Supplement: Supplementary file 19 — Authors’ original file for figure 19 [file 13321_2009_4_MOESM19_ESM.pdf]

```
R=A R*-O#O#O#O#O#O R-A R-O#O#O#O#O#O O#O#O#O#O#O#O-H
 2         2            1        1                  1
```
