# Supplementary material for: A constructive approach for discovering new drug leads: Using a kernel methodology for the inverse-QSAR problem
Source: J Cheminform. 2009 Apr 28;1:4. doi: 10.1186/1758-2946-1-4 (PMC2816860; doi:10.1186/1758-2946-1-4)

```
1) R->A->R->A->R->A->R->O->R*->O->H->O->R*->O->R
2) R->A->R->A->R->A->R->O->R*->O->R*->O->H->O->R
```

1)

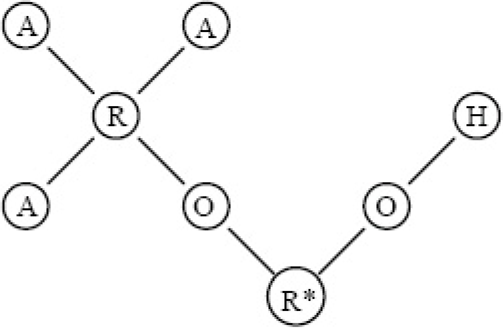

2)

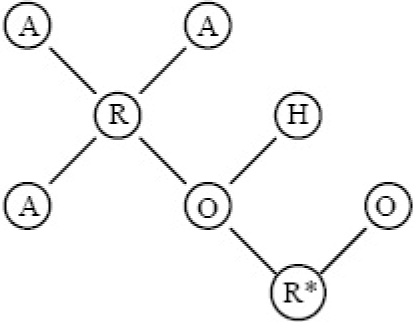

Supplement: Supplementary file 20 — Authors’ original file for figure 20 [file 13321_2009_4_MOESM20_ESM.pdf]
