# Supplementary material for: A constructive approach for discovering new drug leads: Using a kernel methodology for the inverse-QSAR problem
Source: J Cheminform. 2009 Apr 28;1:4. doi: 10.1186/1758-2946-1-4 (PMC2816860; doi:10.1186/1758-2946-1-4)
Supplement: Supplementary file 22 — Authors’ original file for figure 22 [file 13321_2009_4_MOESM22_ESM.pdf]

ACE<br>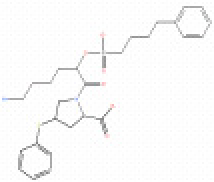<br>Given Activity=9.11<br>Predicted Activity=9.15	Ache<br>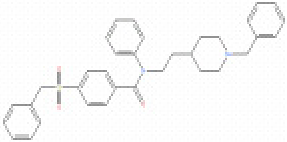<br>Given Activity=9.22<br>Predicted Activity=9.46	BZR<br>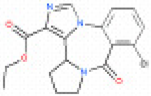<br>Given Activity=8.77<br>Predicted Activity=8.40
COX2<br>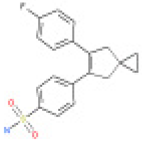<br>Given Activity=8.52<br>Predicted Activity=8.21	DHFR<br>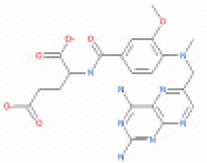<br>Given Activity=8.96<br>Predicted Activity=8.38	GPB<br>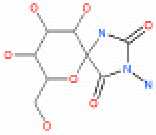<br>Given Activity=6.8<br>Predicted Activity=5.24
THER<br>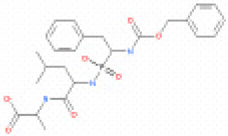<br>Given Activity=10.17<br>Predicted Activity=6.77	THR<br>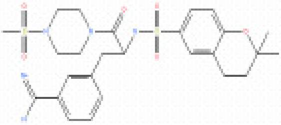<br>Given Activity=8.13<br>Predicted Activity=7.03	
